# The m^6^A regulator KIAA1429 stabilizes RAB27B mRNA and promotes the progression of chronic myeloid leukemia and resistance to targeted therapy

**DOI:** 10.1016/j.gendis.2023.03.016

**Published:** 2023-04-12

**Authors:** Fangyi Yao, Fangmin Zhong, Junyao Jiang, Ying Cheng, Shuai Xu, Jing Liu, Jin Lin, Jing Zhang, Shuqi Li, Meiyong Li, Yanmei Xu, Bo Huang, Xiaozhong Wang

**Affiliations:** Jiangxi Province Key Laboratory of Laboratory Medicine, Jiangxi Provincial Clinical Research Center for Laboratory Medicine, Department of Clinical Laboratory, The Second Affiliated Hospital of Nanchang University, Nanchang, Jiangxi 330006, China

**Keywords:** Chronic myeloid leukemia, KIAA1429, N^6^-methyladenine, RAB27B, Rucaparib, YTHDF1

## Abstract

Chronic myeloid leukemia (CML) is a common adult leukemia. Both the acute phase of the disease and the adverse effects of anti-cancer treatments can lead to a poor prognosis. The N^6^-methyladenine (m^6^A) modification plays an important regulatory role in various physiological and pathological processes. KIAA1429 is a known m^6^A regulator, but the biological role of KIAA1429 in CML is unclear. In this study, we observed that the m^6^A levels and KIAA1429 expression were significantly up-regulated in patients with blast phase CML. Notably, KIAA1429 regulated the total level of RNA m^6^A modification in the CML cells and promoted the malignant biological behaviors of CML cells, including proliferation, migration, and imatinib resistance. Inhibiting KIAA1429 in CML cells reduced the stability of RAB27B mRNA through the m^6^A/YTHDF1 axis, consequently inhibiting CML proliferation and drug efflux, ultimately increasing the sensitivity of CML cells to imatinib. Moreover, the knockdown of RAB27B also inhibited the proliferation and drug resistance of CML cells and promoted their apoptosis. Rucaparib, a recently developed anti-cancer agent, suppressed the expression of KIAA1429 and CML cell proliferation and promoted cell apoptosis. Rucaparib also inhibited the tumorigenesis of CML cells *in vivo*. The combined use of rucaparib and imatinib enhanced the sensitivity of CML cells to imatinib. Our study provides evidence that elevated KIAA1429 expression in the blast phase of CML enhances the stability of RAB27B mRNA through the m^6^A/YTHDF1 axis to up-regulate RAB27B expression, thereby promoting CML progression. Rucaparib exerts inhibitory effects on KIAA1429 expression and thus reduces CML progression.

## Introduction

Chronic myeloid leukemia (CML) is a malignant clonal myeloproliferative disorder originating from myeloid pluripotent hematopoietic stem cells.^1^ The disease is manifested mainly as the abnormal proliferation of mature myeloid cells and accounts for approximately 20% of all adult leukemias. The characteristic genetic signature of CML is the presence of a t (9; 22) translocation leading to the formation of the Philadelphia (Ph) chromosome, which encodes a Bcr/Ab11 fusion protein, an oncoprotein with high tyrosine kinase activity. This has been considered the molecular basis for the development of CML.^2^^,^^3^ The natural course of CML consists of three phases: chronic phase (CP), accelerated phase (AP), and blast crisis (BC). Patients with CML which has progressed to BP have an extremely poor prognosis.^4^ Tyrosine kinase inhibitors (TKIs), represented by imatinib, are targeted therapeutic drugs for CML which have shown high clinical effectiveness.^5^ Unfortunately, approximately 25% of patients eventually develop drug resistance or have poor intrinsic drug responsivity.^6^^,^^7^ The acute phase changes of CML and TKIs resistance are still major challenges in reducing the rate of treatment-free remission (TFR) in CML patients.

In recent years, advances in understanding the roles of epigenetics in the response of tumors to molecular-targeted therapy have provided novel routes for the treatment of CML. Notably, N^6^-methyladenine (m^6^A) modification is the methylation modification of the sixth nitrogen atom of the RNA base adenine (*A*) and is one of the most common modifications of mRNA in eukaryotes.^8^^,^^9^ It has been demonstrated that m^6^A is jointly, reversely, and dynamically regulated by methyltransferases,^10^ demethylases,^11^ and reader proteins.^12^ Methyltransferases add the m^6^A modification to transcripts, whereas demethylases remove the m^6^A modification from transcripts, which are then recognized by reader proteins that exert different effects.^13^ M^6^A modification is important for maintaining the metabolism, stability, splicing, and translation of mRNA. The m^6^A modification regulates various physiological and pathological processes including tumor development, reproduction, and biological rhythms.^14^^,^^15^ Accumulating evidence has also shown that the m^6^A modification regulates the proliferation, differentiation, and metabolism of hematopoietic stem cells and leukemia cells,^16^, ^17^, ^18^ and influences the sensitivity of leukemia cells to chemotherapeutic drugs.^19^^,^^20^

KIAA1429 is one of the major components of the m^6^A methyltransferase complex that mediates the m^6^A modification of coding and non-coding RNA.^21^ KIAA1429 promotes the development of various cancers,^22^, ^23^, ^24^, ^25^ such as breast, liver, and gastric cancers, and is closely associated with a poor prognosis for cancer patients. However, the role of KIAA1429 in CML is still unclear and needs to be further investigated.

In this study, we examined the roles of KIAA1429 in the progression of CML and drug resistance and investigated the underlying mechanisms. Our findings indicate that the KIAA1429/m^6^A/YTHDF1 axis up-regulates the expression of RAB27B by increasing the stability of RAB27B mRNA. Additionally, transcriptome sequencing and RIP-qPCR demonstrated that the KIAA1429/m^6^A/YTHDF1 axis played an important role in the progression of CML. This study aimed to explore the epigenetic mechanisms involved in the progression of CML and to assess the potential value of KIAA1429 as a therapeutic target for CML.

## Methods and materials

### Patients and samples

Peripheral blood mononuclear cells (PBMCs) were isolated from blood specimens from a total of 40 CML-CP and 18 CML-BC patients admitted to the First/Second Affiliated Hospital of Nanchang University from 2018 to 01-01 to 2021-6-30. All patients met the criteria for a diagnosis of chronic phase or blast phase CML, as specified in *The guidelines for the diagnosis and treatment of chronic myelogenous leukemia in China (2020 edition)*. The patients with chronic phase CML were all newly diagnosed and had not received any anti-tumor therapy. All included subjects provided informed consent to participate in the study, and the study protocol was reviewed and approved by the hospital ethics committee.

### Cell culture and transfection

Human chronic myeloid leukemia cell lines K562 and KCL22 were purchased from the American Type Culture Collection (ATCC), and the K562 imatinib-resistant strain, K562/G01, was donated by the School of Laboratory Medicine, Chongqing Medical University (Chongqing, China). The culture conditions were as follows: 37 °C, 5% CO_2_, and saturated humidity. The culture medium consisted of 10% fetal bovine serum (Gibco, USA), 100 U/mL penicillin (Solarbio, China), and 100 U/mL streptomycin (Solarbio, China) in RPMI-1640 (Gibco, USA). We used imatinib (IM) (Apexbio, USA) at a final concentration of 1 μM. Rucaparib (MCE, USA) was diluted to 100 nM in DMSO. Cell lines that had a KIAA1429 (NM_015496.4) overexpression plasmid (Hanbio, China) or were transfected with lentivirus containing shRNA again KIAA1429, RAB27B or YTHDF1 (Genechem, China) were generated (Table S1).

### Quantitative real-time PCR (RT-qPCR)

Total RNA was extracted by using the Trizol reagent (Invitrogen, USA). First-strand cDNA was synthesized with the PrimeScript™ Reverse Transcriptase kit (Takara, Japan) and random primers. Real-time quantitative PCR was performed with a TB Green® Premix EX TaqTMⅡ qPCR Master Mix (Takara, Japan). All reactions were run in triplicate on the Applied Biosystems 7500 Real-Time PCR System. GAPDH was used as an endogenous control. The relative expression was calculated using the comparative Ct (2^−ΔΔCT^) method. The sequences of the primers are listed in Table S2.

### Western blotting

Cells were lysed in RIPA lysis buffer (Applygen, China). The protein concentration was detected with a bicinchoninic acid (BCA) kit (Tiangen, China). During electrophoresis, 40 ng of protein was added to each well and run on 8% SDS-PAGE. The proteins in the gel were then transferred onto PVDF membranes (Millipore, Germany). The membranes were blocked with 5% nonfat milk and incubated for 2 h at room temperature. An anti-GAPDH (#2218 S, Cell Signaling Technology, 1:1000), anti-KIAA1429 (#51104, CST, 1:1000), anti-RAB27B (AO0173, Boster, 1:1000), or anti-YTHDF1 (#51104, CST, 1:1000) was then added, followed by overnight incubation at 4 °C. After being rinsed three times, the secondary antibody (Anti-Rabbit, 7074 S, CST, 1:5000) was added, followed by incubation for 2 h at room temperature. Finally, blots were detected using an enhanced chemiluminescence kit (Thermo, USA) and analyzed by a Bio-Rad Western Blot Imaging System (Bio-Rad, Berkeley, CA, USA).

### The CCK-8 cell proliferation assay

The proliferation of each cell line was examined using the Cell Counting Kit-8 (Hambio, China), following the manufacturer's instructions. Briefly, 100 μL of 1 × 10^5^/mL cells were seeded into 96-well plates. The cells were then cultured in a cell incubator. A volume of 10 μL of CCK-8 solution was added into each well 0, 12, 24, and 48 h later. Following incubation at 37 °C for another 2 h, the optical density (OD) values at 450 nm were measured using a microplate reader (PERLONG, China).

### EdU assay

Cell proliferation was detected by EdU staining. As suggested by the instructions provided with the reagent (RIBOBIO, China), an aliquot of 1 × 10^6^ cells in the logarithmic growth phase was taken and incubated for 2.5 h with EdU solution diluted 1:1000 in a complete medium. After fixation in polyformaldehyde at room temperature, each of the samples was decolorized with 2 mg/mL glycine. Apollo staining and Hoechst33342 chamber staining were performed in sequence, and after washing, fluorescence microscopy was used for the determination of cell proliferation.

### Flow cytometry

A total of 1 × 10^5^ cells were collected and washed with pre-cooled PBS. Then, 5 μL of Annexin V-APC (BD, USA) and 5 μL of 7-AAD staining solution were added into each of the tubes, followed by incubation in the dark for 10 min at room temperature. After adding binding buffer, flow cytometry analyses were performed within 1 h using a BD FACS Calibur Flow Cytometer (BD, San Diego, CA, USA).

### Migration assays

Transfected cells were starved in a serum-free medium for 24 h. Cells were then added to Transwell chambers at a density of 1 × 10^6^/mL. After 48 h of culture, the chamber was taken out, immersed in 4% paraformaldehyde for 30 min, and stained with 0.1% crystal violet for approximately 30 min. The cells were washed with PBS, and cell migration was determined by counting the cells which had migrated across the chamber under an inverted microscope.

### Half maximal inhibitory concentration

Cells in the logarithmic growth stage were selected and the concentration of the cell suspension was adjusted to 1 × 10^5^/mL. Subsequently, a 100 μL aliquot of the cell suspension was added to each well of a 96-well plate, and different concentration gradients of therapeutic drugs were added to the wells. All concentrations were tested in triplicate. A blank complete medium was used to establish the baseline. After the 96-well plates were maintained in a constant temperature cell incubator for 48 h, 10 μL of CCK-8 solution was added to each well, and the plates were incubated for an additional 2–4 h. Finally, the absorbance value was detected with 450 nm as the detection wavelength and 630 nm as the reference wavelength for the enzyme label, and the IC_50_ curve was drawn to assess the impact of the different treatments.

### Assessment of RNA stability

Cells in the logarithmic growth phase were resuspended to 1 × 10^5^/mL in serum-free medium, and 5 μg/mL actinomycin D was added. Each group of cells was transferred into four parallel wells, corresponding to four time points. The cells were collected after 0, 2, 4, and 6 h of incubation at a constant temperature in a cell incubator. Total mRNA was extracted from each group of cells by the Trizol method, and the target gene expression was detected by RT-qPCR. Further, result curves were drawn to observe the changes in mRNA expression of different target genes, and the degradation rates were compared to assess the mRNA stability.

### Total RNA m^6^A modification level

The m6A level of RNA was detected by an ELISA method. First, the sample RNA was treated with a gDNA wiper mix (Vazyme, China). PolyA^+^ mRNA was purified using a Dynabeads™ mRNA Purification Kit (Invitrogen, USA). An EpiQuik m^6^A RNA Methylation Quantification Kit (Colorimetric) (Epigentek, USA) was utilized to measure the global m^6^A levels in mRNA following the manufacturer's protocol. A total of 200 ng of polyA^+^ mRNA was used for each sample analysis. The absorbance (OD) value was measured using 450 nm as the detection wavelength and the standard curve was drawn to calculate the m6A level.

### mRNA sequencing

Total RNA was extracted from K562 cells with KIAA1429 silenced, and the samples from the control group were subjected to extraction with the TRIzol reagent. The library preparations were sequenced by Seqhealth Technology Co., Ltd. (Wuhan, China). Gene ontology terms were considered significantly differentially expressed for samples with an adjusted *P* < 0.05 and a fold-change >1.

### RNA immunoprecipitation qPCR (RIP)

RIP measurements were conducted using the Magna RIP kit (Millipore, MA, USA). In brief, K562 cells were lysed with RIP Lysis Buffer. Next, the RIP lysate supernatant and the specific antibody were incubated overnight on protein A/G magnetic beads at 4 °C. After being washed, total RNA was extracted with TRIzol, and quantitative RT-qPCR analysis was performed.

### Liquid chromatography-mass spectrometry (LC-MS)

Imatinib concentrations were detected by LC-MS. After the cells were treated with imatinib for 48 h, the medium was replaced and cells were cultured for another 24 h. The extracellular (culture media) and intracellular cell lysates were collected. The internal standard solution was diluted 100 times with methanol to prepare a precipitant. Samples were aspirated into centrifuge tubes, and the precipitant containing the internal standard was added to each tube. The tubes were then vortex-centrifuged, and the supernatants were collected and diluted with purified water, followed by chromatographic analysis. Quantitative processing of sample data using the internal standard method and multi-point calibration curve quantification was conducted.

### SELECT detection

Total RNA, polyA-RNA, or synthesized RNA oligos were mixed with primers and dTTP in CutSmart buffer. The RNA and primers were annealed in an incubation mixture at a temperature gradient as follows: 90 °C for 1 min, 80 °C for 1 min, 70 °C for 1 min, 60 °C for 1 min, 50 °C for 1 min, and then 40 °C for 6 min. Subsequently, a mixture containing DNA polymerase, SplintR ligase, and ATP was added. The final reaction mixture was incubated at 40 °C for 20 min, denatured at 80 °C for 20 min, and kept at 4 °C until quantitative real-time PCR (qPCR) was performed. The qPCR was run under the following conditions: 95 °C for 5 min (95 °C, 10 s; 60 °C, 35 s) × 40 cycles; 95 °C for 15 s; 60 °C for 1 min; and 95 °C for 15 s; 4 °C. Data were analyzed with QuantStudioTM Real-Time PCR Software Version 1.3.

### *In vivo* study

Female BALB/C-nu mice were selected as experimental animals. In the toxicity experiments, nude mice were given 50 mg/kg/d rucaparib that dissolved in saline via intragastric administration; the control group was administered an equal volume of normal saline. After one week of treatment, the mice were anesthetized, and the liver, spleen, kidney, tumor tissue, and blood were sampled, followed by euthanasia for the mice. In the therapeutic trials, the nude mice were used to create subcutaneous tumorigenesis models. In brief, K562 cells (1 × 10^6^) were suspended in 100 μL of normal saline and the suspension was mixed with an equal volume of Matrigel. This mixture was subcutaneously injected into the right armpit of 4-week-old mice. Tumor size measurements were initiated on the day of inoculation. The tumor size was calculated using the formula: 0.5 × (long diameter) × (short diameter).^2^ When the tumor volume reached 100 mm^3^ (± 20%), rucaparib was given via intragastric administration at 50 mg/kg/d; the control group was given the same volume of normal saline. After 2–4 weeks, the mice were anesthetized, and the liver, spleen, kidney, tumor tissue, and blood were sampled, followed by euthanasia for the mice. All animal experiments were approved by the Animal Protection and Use Committee of the Second Affiliated Hospital of Nanchang University (Nanchang, China).

### Statistical analysis

Statistical analysis was performed using the SPSS 20.0 and GraphPad statistical software programs. The values of the results were expressed as the means ± standard deviation of at least three independent experiments. Data were analyzed using Student's *t*-test and ANOVA. A *P*-value < 0.05 was considered statistically significant.

## Results

### KIAA1429 expression is up-regulated in CML-BC patients

**Figure 1:**
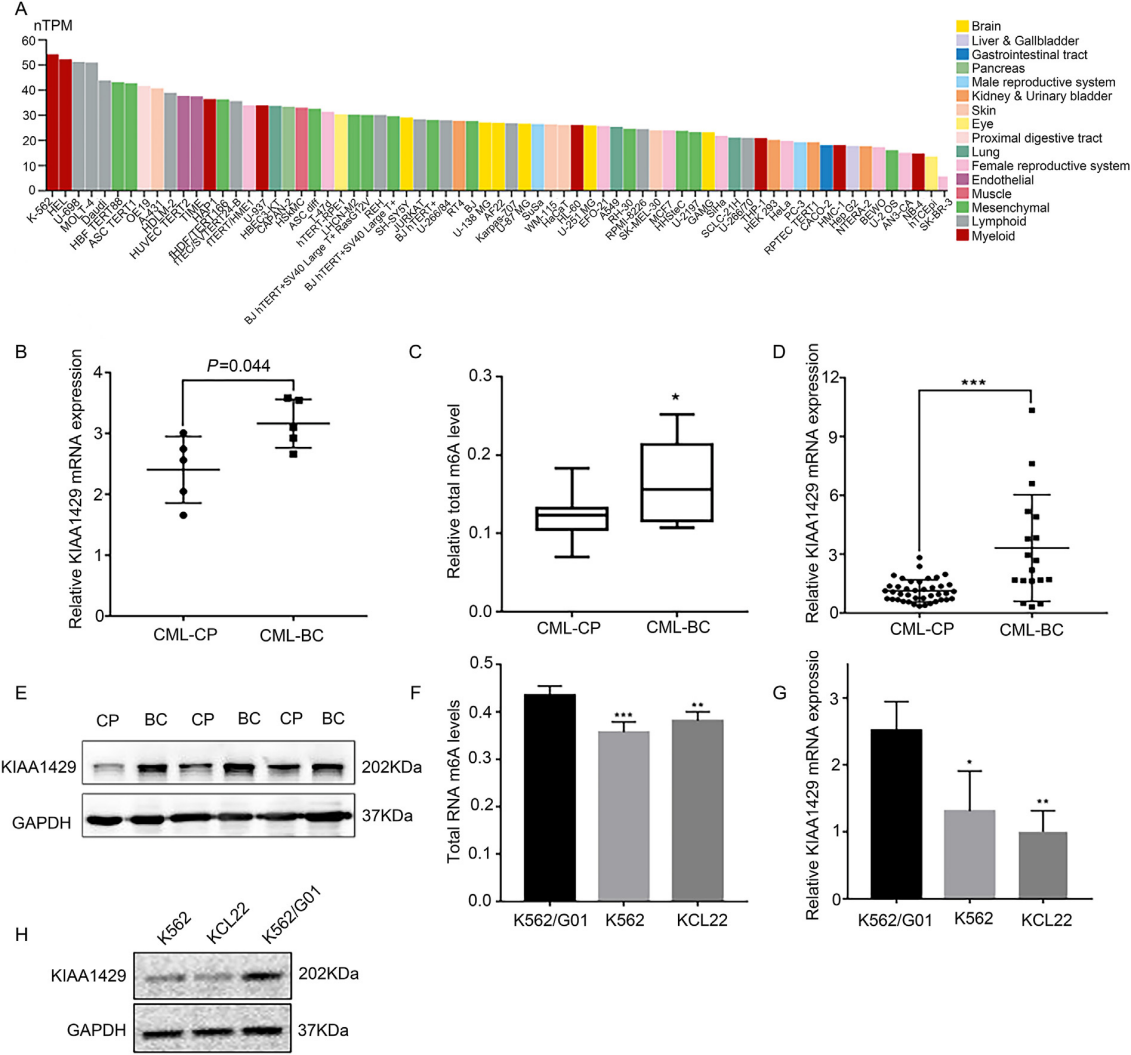
The KIAA1429 expression and m^6^A modification level were up-regulated in chronic myeloid leukemia. **(A)** The HPA database showed that the KIAA1429 mRNA expression in K562 cells was the highest in all examined cell lines. **(B)** The average expression level of KIAA1429 mRNA in CML chronic phase (CP) patients and blast crisis (BC) patients, based on data from the GEO database (GSE100026). **(C)** A colorimetric method was used to detect the level of total RNA m^6^A modification in CML-BC patients and CML-CP patients. **(D)** RT-qPCR was used to detect the KIAA1429 mRNA expression levels in CML-BC patients (*n* = 18) and CML-CP patients (*n* = 40). **(E)** Western blotting was used to detect the KIAA1429 protein expression levels in CML-BC patients and CML-CP patients. **(F)** A colorimetric method was used to detect the level of total RNA m^6^A modification in different CML cell lines. **(G)** RT-qPCR was used to detect the KIAA1429 mRNA expression levels in different CML cell lines. **(H)** Western blotting was used to detect the KIAA1429 protein expression in different CML cell lines. ^∗^*P* < 0.05, ^∗∗^*P* < 0.01, ^∗∗∗^*P* < 0.001.

**Fig. S1:**
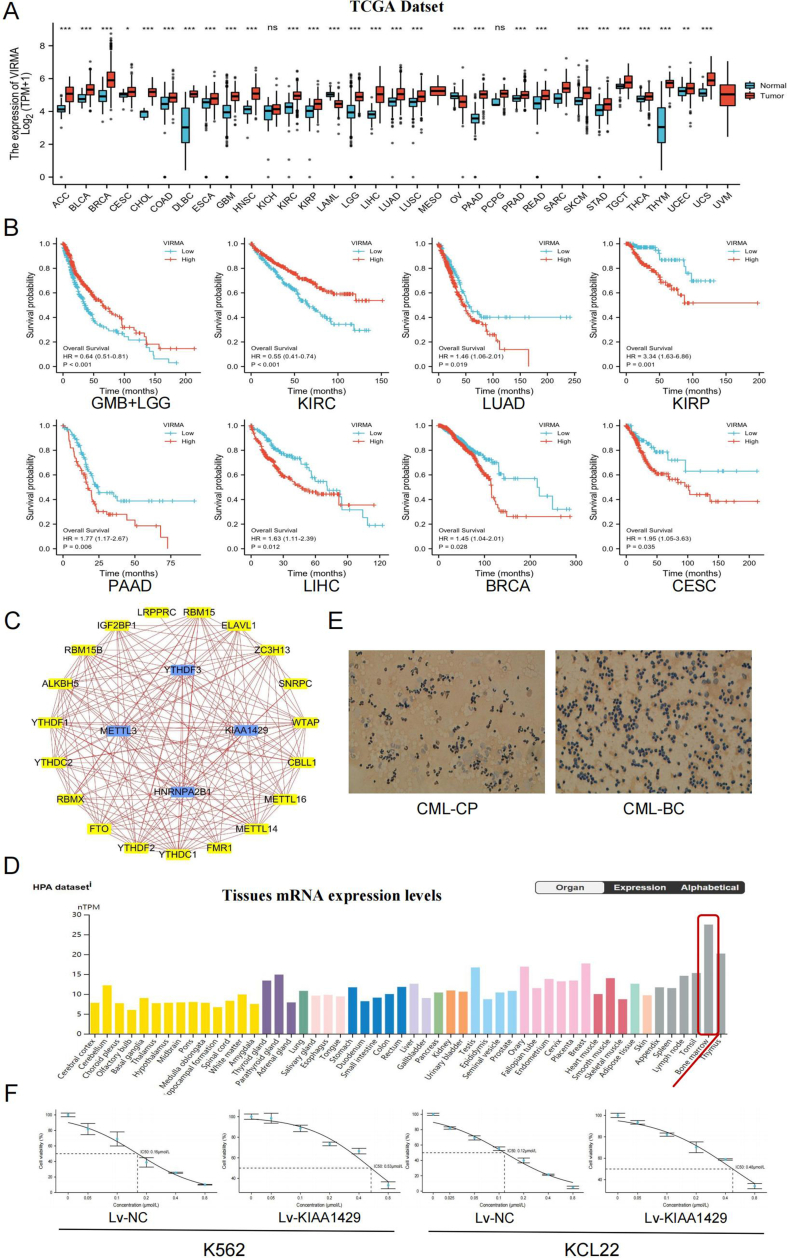
Pan-cancer analysis of KIAA1429 expression. **(A)** The KIAA1429 mRNA expression in a variety of cancer cells, based on data from the TCGA database. **(B)** Assessment of the relationship between the KIAA1429 expression level and overall survival rate of cancer patients as indicated by a Kaplan–Meier curve. **(C)** A PPI network was constructed using 23 major genes associated with the m^6^A modification. **(D)** The HPA database showed that the KIAA1429 mRNA expression was the highest in bone marrow compared to other body tissues. **(E)** IHC was used to evaluate the KIAA1429 protein expression level in the bone marrow for CML-BC patients and CML-CP patients. **(F)** The IC50 of imatinib was detected in K562 and KCL22 cells by a cytotoxicity assay after the overexpression of KIAA1429. ∗*P <* 0.05, ∗∗*P <* 0.01, ∗∗∗*P* < 0.001.

To explore the function of KIAA1429, the expression data were analyzed for 16 cancer cell lines documented in the Human Protein Atlas (HPA) database. The expression of m^6^A-related methyltransferase, KIAA1429, was found to be dysregulated in a variety of tumors (Fig. S1A) and its expression was closely related to tumor progression and a poor prognosis (Fig. S1B). Importantly, KIAA1429 was found to be a key regulator of the m^6^A modification (Fig. S1C). The findings also showed that, in comparison with other cancers, the expression of KIAA1429 was the highest in a CML cell line (Fig. 1A). In addition, of all human tissues, bone marrow had the highest expression of KIAA1429 (Fig. S1D).

Our team also analyzed the sequencing data for 40 patients with CML-CP and 18 patients with CML-BC (data were deposited under accession number GSE100026). Our findings showed that the expression of m^6^A writer KIAA1429 was up-regulated in CML-BC compared with CML-CP (Fig. 1B). We also found that the total m^6^A modification level (Fig. 1C) and KIAA1429 expression (Fig. 1D, E; Fig. S1E) were significantly more up-regulated in the CML-BC group than that in the CML-CP group, indicating that KIAA1429 could be associated with CML progression.

Imatinib is a first-line drug for the treatment of CML, and the progression of CML is generally associated with imatinib resistance. To examine their possible relationship with imatinib resistance, the m^6^A modification level and KIAA1429 expression were measured in the K562 and KCL22 CML cell lines, as well as in the imatinib-resistant K562 cell line (K562/G01). The results revealed that the total m^6^A modification level (Fig. 1F), as well as the mRNA and protein levels of KIAA1429 (Fig. 1G, H), were significantly up-regulated in K562/G01 cells compared with the K562 and KCL22 cells. In summary, KIAA1429 could up-regulate the m^6^A modification, which was associated with increased imatinib resistance and tumor progression in CML.

### Increased KIAA1429 expression promotes the malignant characteristics of CML cells

**Figure 2:**
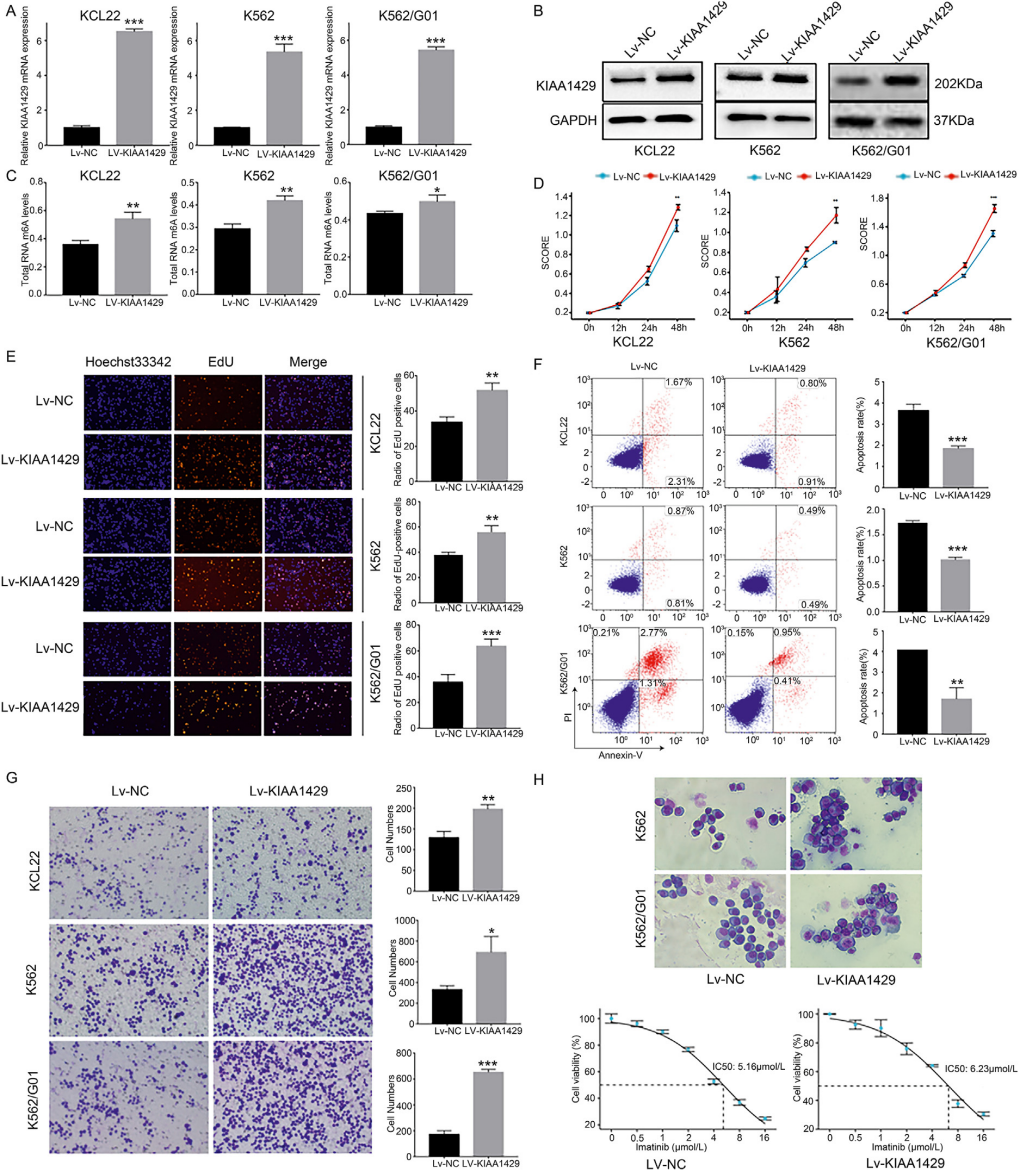
KIAA1429 up-regulation promoted the malignant characteristics of CML cells. **(A)** RT-qPCR was used to verify the efficiency of KIAA1429 overexpression at the mRNA level. **(B)** Western blotting was used to verify the efficiency of KIAA1429 overexpression at the protein level. **(C)** A colorimetric method was used to detect the changes in the total RNA m^6^A modification level in CML cells after the overexpression of KIAA1429. **(D)** CCK-8 assays were used to detect the viability of CML cells after the overexpression of KIAA1429. **(E)** EdU staining was used to detect the proliferation of CML cells after the overexpression of KIAA1429. **(F)** Flow cytometry was used to detect the apoptosis of CML cells after the overexpression of KIAA1429. **(G)** Transwell assays were used to detect the migration of CML cells after the overexpression of KIAA1429. **(H)** The morphology of CML cells was observed by Reisher-Giemsa staining after the overexpression of KIAA1429. **(I)** The IC_50_ of imatinib was detected in K562/G01 CML cells using a cytotoxicity assay after the overexpression of KIAA1429. ^∗^*P* < 0.05, ^∗∗^*P* < 0.01, ^∗∗∗^*P* < 0.001.

To investigate the biological effects of KIAA1429 in CML, a KIAA1429 overexpression plasmid, Lv-KIAA1429, was constructed and used to transfect CML cell lines (K562, K562/G01, and KCL22) (Fig. 2A, B). Our results showed that the total m^6^A modification level in the CML cells with Lv-KIAA1429 was significantly higher than that in the control cells (Lv-NC) (Fig. 2C). It was also observed that KIAA1429 overexpression enhanced the proliferation and migration capabilities of CML cells (Fig. 2D, E, G), while the cell apoptosis rate decreased (Fig. 2F). The Wright-Giemsa staining of cells before and after the overexpression revealed a higher number of undifferentiated cells in the cell lines with KIAA1429 overexpression than in the control cells. In addition, the percentages of promyelocytes and myelocytes were increased, and the morphological characteristics were similar to the bone marrow characteristics that occur during the transformation of CML from the chronic phase to the acute phase (Fig. 2H).

The cell viability assay showed that the resistance of K562/G01 cells to imatinib was significantly up-regulated (Fig. 2I), overexpression of KIAA1429 also increase imatinib resistance in the CML IM sensitive cell line K562 and KCL22 (Fig. S1F), suggesting that the increased expression of KIAA1429 may be associated with the imatinib resistance of CML cells. In summary, these findings showed that elevated expression of KIAA1429 reduced the sensitivity of CML cells to imatinib and inhibited the differentiation of CML cells, which could be a potential mechanism underlying the acute transformation of CML.

### Knocking down KIAA1429 expression reduces the malignant characteristics of CML cells

**Figure 3:**
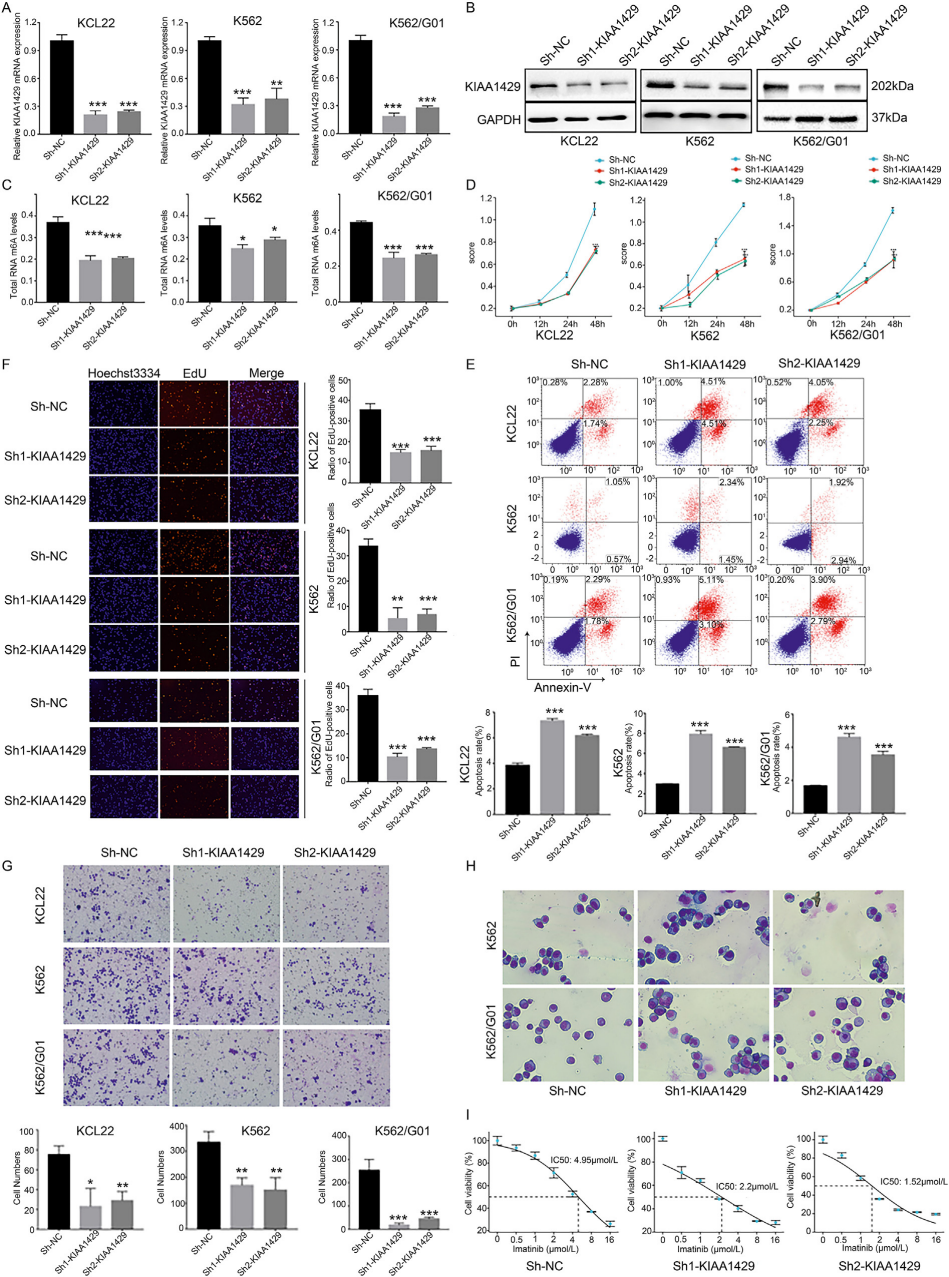
KIAA1429 down-regulation reduces the malignant characteristics of CML cells. **(A)** RT-qPCR was used to verify the efficiency of KIAA1429 knockdown at the mRNA level. **(B)** Western blotting was used to verify the efficiency of KIAA1429 knockdown at the protein level. **(C)** A colorimetric method was used to detect the changes in the total RNA m^6^A modification level in CML cells after KIAA1429 knockdown. **(D)** CCK-8 assays were used to detect the viability of CML cells after the knockdown of KIAA1429. **(E)** EdU staining was used to detect the proliferation of CML cells after KIAA1429 knockdown. **(F)** Flow cytometry was used to detect the apoptosis of CML cells after the knockdown of KIAA1429. **(G)** Transwell assays were used to detect the migration of CML cells after the knockdown of KIAA1429. **(H)** The morphology of CML cells was observed by Reisher-Giemsa staining after KIAA1429 knockdown. **(I)** The IC_50_ of imatinib was determined in K562/G01 cells by cytotoxicity assays after KIAA1429 was knocked down. ^∗^*P* < 0.05, ^∗∗^*P* < 0.01, ^∗∗∗^*P* < 0.001.

The effects of KIAA1429 knockdown on CML progression were investigated to further determine how KIAA1429 impacts the malignant characteristics of the cells. First, a stable CML cell line with KIAA1429 knockdown (Sh-KIAA1429) was constructed (Fig. 3A, B). The KIAA1429 knockdown reduced the total level of RNA m^6^A modification in CML cells (Fig. 3C). Notably, CML cell proliferation was inhibited (Fig. 3D, E), cell apoptosis was increased (Fig. 3F), and cell migration was suppressed (Fig. 3G). Morphological observations showed that KIAA1429 knockdown increased the percentage of lobulated or stab-nuclear (curved/curled, but not lobulated, nucleus) cells, and the cells were stabilized in the chronic phase (Fig. 3H). Moreover, KIAA1429 knockdown significantly reduced the imatinib resistance of K562/G01 cells (Fig. 3I). These findings demonstrated that KIAA1429 knockdown could reduce the malignant biological characteristics of CML.

### *RAB27B* is the downstream target of KIAA1429

**Figure 4:**
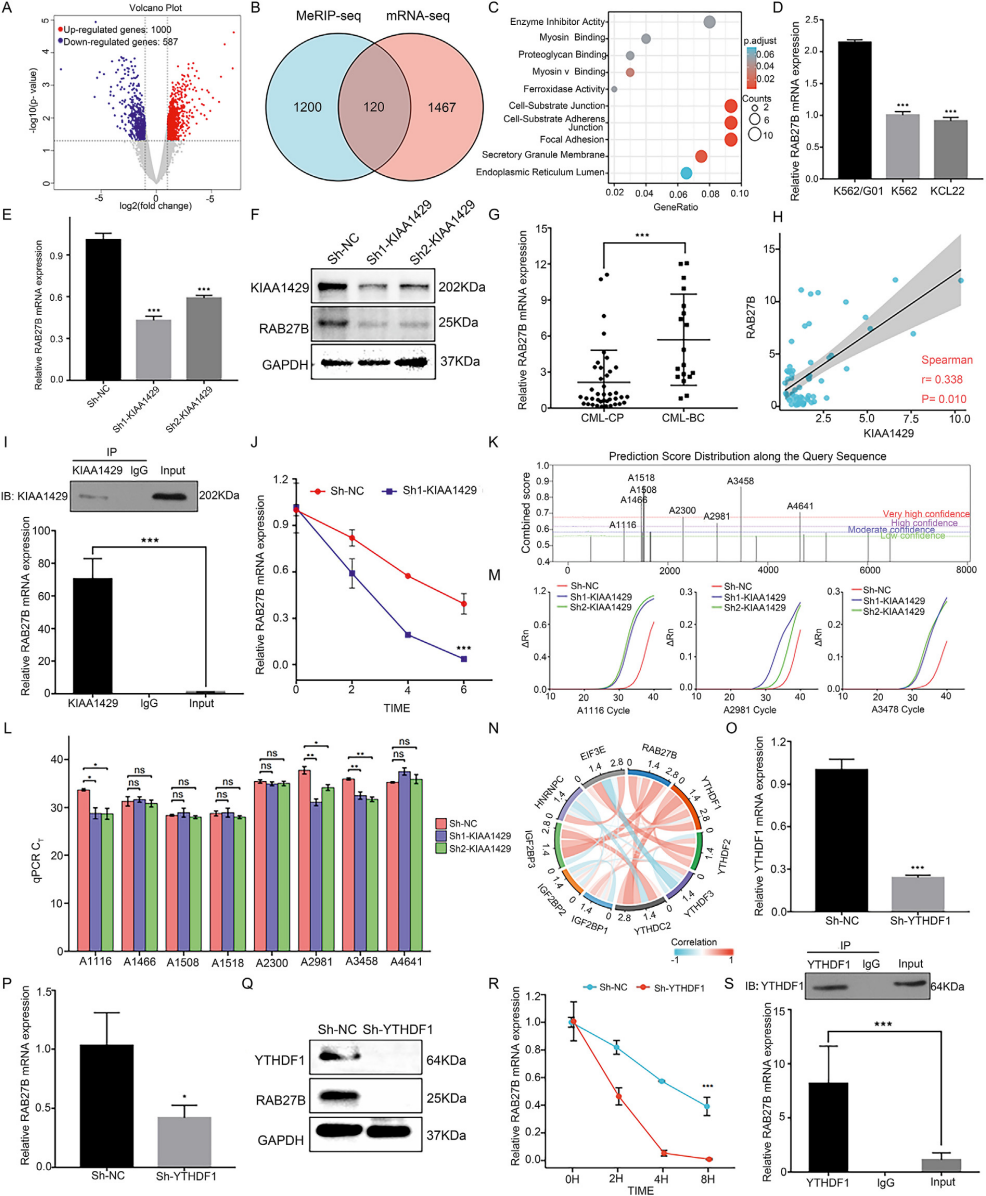
RAB27B is the downstream target gene of KIAA1429. **(A)** Volcano plot represented the changes in the distribution of mapped transcripts after KIAA1429 was knocked down in K562 cells. **(B)** An intersection Venn diagram of the meRIP-seq and mRNA-seq data. **(C)** A bubble diagram of the genes that intersected from the GO and KEGG enrichment analysis. **(D)** RT-qPCR was used to detect the RAB27B mRNA expression levels in different CML cell lines. **(E)** RT-qPCR was used to detect the RAB27B mRNA expression level after KIAA1429 knockdown. **(F)** Western blotting was used to detect the KIAA1429 and RAB27B protein expression levels after KIAA1429 knockdown. **(G)** RT-qPCR was used to analyze the RAB27B mRNA expression level in CML-BC patients (*n* = 18) and CML-CP patients (*n* = 40). **(H**) The correlation between the KIAA1429 and RBA27B mRNA expression levels in CML patients. **(I)** The results of a RIP-qPCR analysis of the interaction between the KIAA1429 protein and RAB27B mRNA. **(J)** An mRNA stability assay was used to detect the effects of KIAA1429 knockdown on RAB27B mRNA stability. **(K)** Prediction of the RAB27B m^6^A modification sites, based on data from the SRAMP website. **(L)** SELECT was used to detect the m^6^A modification sites in RAB27B. **(M)** qPCR quantification of the A1116, A2981, and A3478 m^6^A modification sites in the transcripts from the total RNA obtained from Sh-NC and Sh-KIAA1429 samples. **(N)** Chord diagram representing the coexpression of RAB27B and m^6^A reader proteins, based on the GSE100026 dataset from the GEO database. **(O)** RT-qPCR was used to detect the YTHDF1 mRNA expression level after YTHDF1 knockdown. **(P)** RT-qPCR was used to detect the RAB27B mRNA expression level after YTHDF1 knockdown. **(Q)** Western blotting was used to detect the YTHDF1 and RAB27B protein expression levels after YTHDF1 knockdown. **(R)** An mRNA stability assay was used to detect the effects of YTHDF1 knockdown on RAB27B mRNA stability. **(S)** The results of a RIP-qPCR analysis of the interaction between the YTHDF1 protein and RAB27B mRNA. ∗*P* < 0.05, ∗∗*P* < 0.01, ∗∗∗*P* < 0.001.

**Fig. S2:**
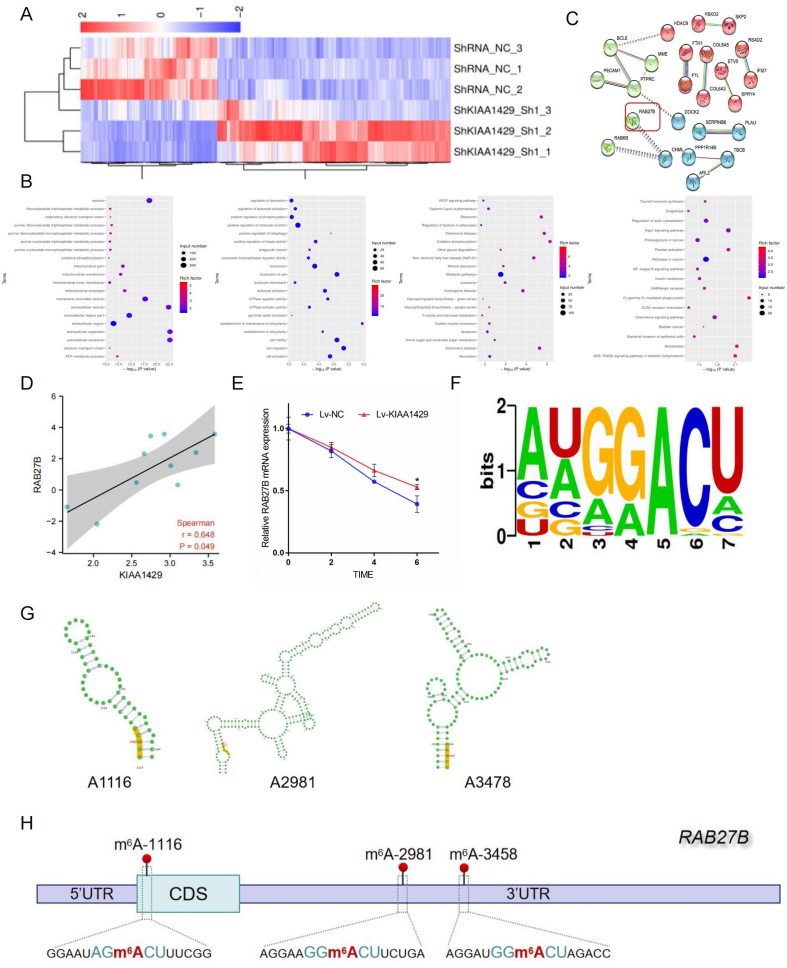
Bioinformatic analysis. **(A)** A heatmap representing the changes in the distribution of mapped transcripts after KIAA1429 was knocked down in each sample of K562 cells. **(B)** GO and KEGG enrichment analyses are shown in bubble diagrams. **(C)** A PPI network was constructed using 120 intersecting genes. **(D)** The correlation between the KIAA1429 and RBA27B mRNA expression levels in CML patients based on the GSE100026 dataset. **(E)** The mRNA stability assay was used to detect the effects of KIAA1429 up-regulation on RAB27B mRNA stability. **(F)** The highest frequency m^6^A modification motif sequences. **(G)** The m^6^A modification structure diagram. **(H)** A schematic diagram of the distribution of RAB27B m^6^A modification sites. ∗*P <* 0.05, ∗∗*P <* 0.01, ∗∗∗*P* < 0.001.

KIAA1429 is the critical enzyme for the m^6^A methylation modification, and its dysregulation induces the activation of relevant cancer pathways. In this study, the KIAA1429 expression was knocked down by shRNA in K562 cells, and K562-Sh1 and K562 parental cells were subjected to an mRNA-seq analysis. The screening was performed using |logFC| >1 and *P*-value <0.05 as the criteria, which retrieved 1587 differentially expressed genes (DEGs) (Fig. 4A; Fig. S2A, B). To clarify the association between these DEGs and the m^6^A modification, the KIAA1429-related MeRIP-seq data in the m6a2target database (http://m6a2target.canceromics.org/#/perturbation) were differentially analyzed (|logFC| > 1 and *P*-value < 0.05), and 1499 m^6^A-related DEGs were retrieved. An OVERLAP analysis was performed for the two groups of DEGs, and 120 co-expressed DEGs (cDEGs) were identified (Table S3 and Fig. 4B). Gene Ontology (GO) and Kyoto Encyclopedia of Genes and Genomes (KEGG) analyses were performed for the cDEGs, which showed that the biological functions of the genes were mainly enriched in enzyme activity inhibition, focal adhesion, and exocytosis (Fig. 4C).

A network analysis of protein–protein interactions (PPIs) (https://cn.string-db.org/) showed that *RAB27B* is the hub gene of the network (Fig. S2C), indicating that *RAB27B* might be a key target gene regulated by KIAA1429. A subsequent RT-qPCR analysis showed that the expression of *RAB27B* mRNA in K562/G01 cells was significantly higher than that in K562 and KCL22 cells (Fig. 4D); while in K562 cells with KIAA1429 knockdown, the *RAB27B* level also decreased with the reduction of the KIAA1429 level (Fig. 4E, F). In clinical samples of CML, the expression of *RAB27B* mRNA was significantly higher in the CML-BC group than in the CML-CP group (Fig. 4G). In addition, the *RAB27B* expression was significantly positively associated with the KIAA1429 expression (*r* = 0.338, *P* = 0.010) (Fig. 4H). This trend was also observed in the GSE100026 dataset (*r* = 0.648, *P* = 0.049) (Fig. S2D). These findings suggest that *RAB27B* is an important downstream target gene for KIAA1429.

### KIAA1429 regulates *RAB27B* mRNA stability in an m^6^A-dependent manner and can be recognized by YTHDF1

RNA immunoprecipitation (RIP)-qPCR was utilized to further assess the relationship between KIAA1429 and *RAB27B*. After enrichment using a KIAA1429-antibody, the amount of *RAB27B* mRNA detected increased significantly, indicating that *RAB27B* mRNA binds to KIAA1429 (Fig. 4I). After KIAA1429 was knocked down, CML cells were treated with actinomycin D to inhibit RNA synthesis. We found that the *RAB27B* mRNA expression level decreased faster over time in the Sh1-KIAA1429 group (Fig. 4J; Fig. S2E), indicating that KIAA1429 regulated the stability of RAB27B mRNA and consequently influenced the expression and function of RAB27B.

The m^6^A methylation sites of RAB27B mRNA were predicted using the SRAMP website (http://www.cuilab.cn/sramp), which showed that eight m^6^A modification sites in the sequences of RAB27B mRNA, *i.e.*, A116, A1466, A1508, A1518, A2300, A2981, A3478, and A4641, had a relatively high possibility of modification (Fig. 4K; Fig. S2F). The SELECT assay was performed to measure the m^6^A modification levels at the sites before and after knockdown and showed that after KIAA1429 knockdown, the m^6^A modifications at the A1116, A2981, and A3478 sites decreased significantly (Fig. 4L, M; Fig. S2G, H), while the modifications at other sites did not, indicating that KIAA1429 knockdown reduces the m^6^A modification level at these sites.

Previous studies have revealed that m^6^A reader proteins could recognize the m^6^A modification in the mRNA of transcripts and consequently regulate the expression of target mRNAs, influencing the biological processes in cancer cells. To further explore the molecular mechanisms related to RAB27B m^6^A modification, the relevant reader proteins were searched based on bioinformatic measurements. Of the various m^6^A reader proteins, YTHDF1 was positively correlated with the levels of RAB27B mRNA (GEO: GSE100026) (Fig. 4N), indicating that YTHDF1 could be a key reader protein for RAB27B.

This finding was confirmed by *in vitro* experiments. We constructed K562 sh-YTHDF1 cells using a lentivirus (Fig. 4O). Our results showed that after the knockdown of YTHDF1, the mRNA and protein levels of RAB27B decreased (Fig. 4P, Q), and the stability of *RAB27B* mRNA also decreased (Fig. 4R). RIP-qPCR findings showed that *RAB27B* mRNA could be enriched by the YTHDF1 protein, and YTHDF1 could influence the stability of RAB27B mRNA (Fig. 4S). These findings indicate that YTHDF1 can bind to *RAB27B* mRNA in CML cells.

### Inhibiting RAB27B expression rescued the promotion of CML cell proliferation and drug resistance by KIAA1429

**Figure 5:**
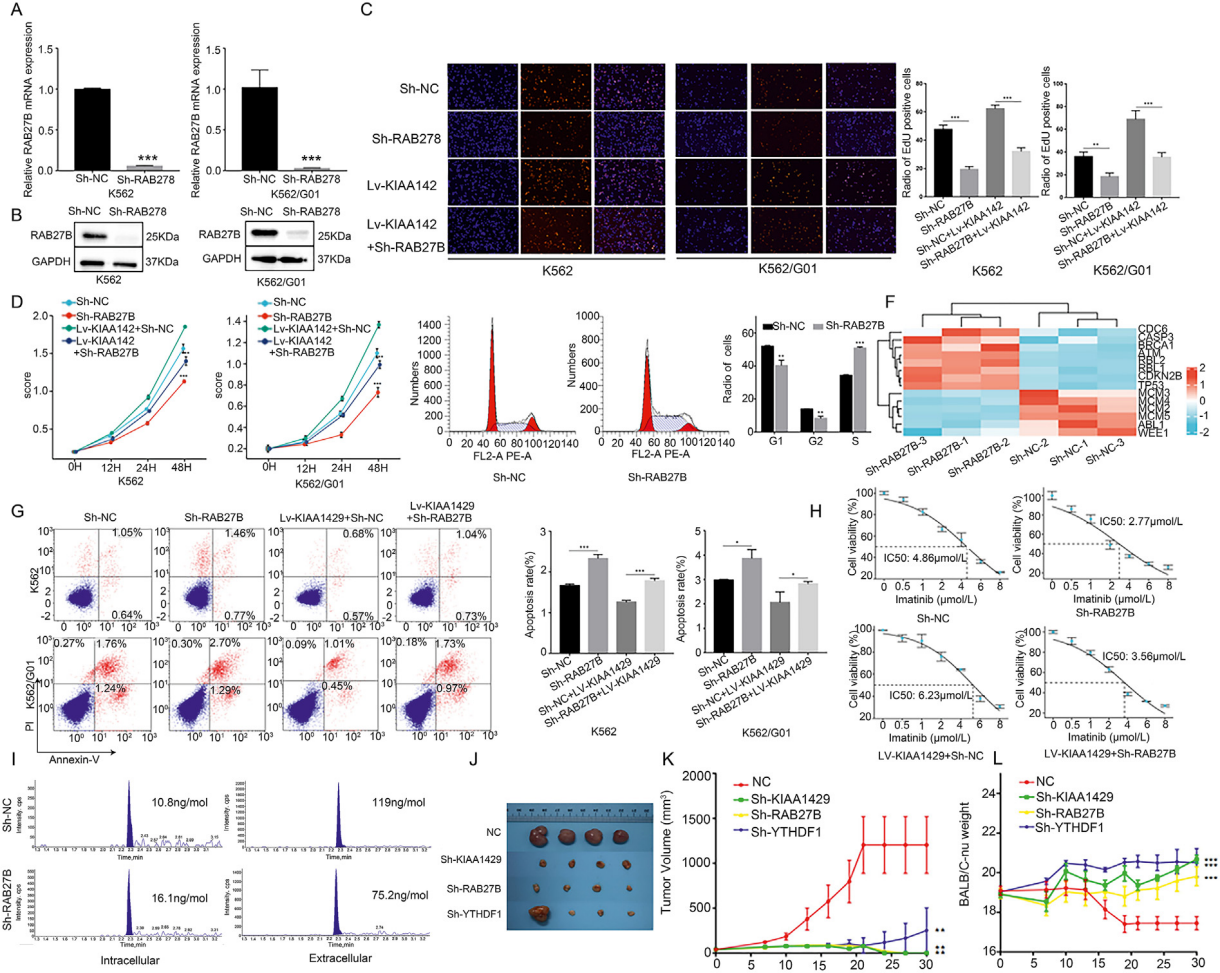
RAB27B promoted the malignant characteristics of CML cells, and inhibiting RAB27B expression rescued CML cells from KIAA1429-induced proliferation and drug resistance. **(A)** RT-qPCR was used to verify the efficiency of RAB27B knockdown at the mRNA level. **(B)** Western blotting was used to verify the efficiency of RAB27B knockdown at the protein level. **(C)** The proliferation of CML cells was determined using EdU staining. **(D)** CCK-8 assays were used to detect the viability of CML cells. **(E)** Cell cycle assays were used to evaluate the cell cycle progression. **(F)** Pathway arrays were used to detect the changes in the expression of cell cycle-related genes. **(G)** Flow cytometry was used to detect the apoptosis of CML cells. **(H)** The IC_50_ of imatinib in K562/G01 cells was determined by a cytotoxicity assay. **(I)** The concentration of imatinib in cells and cell culture medium was detected by LC-MS. **(J)** Images of tumors derived from each group of cells in BALB/C-nu mice. **(K)** Body weight gain curves of the BALB/C-nu mice in each group. **(L)** Tumor volume growth curve of the BALB/C-nu mice in each group. ^∗^*P* < 0.05, ^∗∗^*P* < 0.01, ^∗∗∗^*P* < 0.001.

Previous studies have shown that RAB27B could regulate cell signal transduction and exosome release, and thus influence tumor proliferation and drug resistance. However, the role of RAB27B in CML is still unclear. To clarify the biological functions of RAB27B in CML, K562, and K562/G01 parental cells and cells with KIAA1429 overexpression were transfected with the lentivirus to knock down RAB27B (Fig. 5A, B). After the RAB27B knockdown, the proliferation capability of the parental and KIAA1429-overexpressing K562 and K562/G01 cells decreased (Fig. 5C, D), although the KIAA1429 overexpression partially rescued the inhibitory effects of RAB27B knockdown on cell proliferation.

Cells were arrested in the S phase after RAB27B knockdown (Fig. 5E). Pathway arrays showed that the expression levels of genes associated with the S phase, such as *ABL1* and *MCM2*, were lower, whereas the levels of negative regulatory genes, such as *CDKN2B* and *TP53*, were higher (Fig. 5F). RAB27B knockdown induced the up-regulation of cell apoptosis and suppressed the inhibitory effects of KIAA1429 overexpression on CML cell apoptosis (Fig. 5G). In addition, RAB27B knockdown increased the sensitivity of K562/G01 cells to imatinib (Fig. 5H), while KIAA1429 overexpression enhanced the resistance of cells with RAB27B knockdown to the drug.

**Fig. S3:**
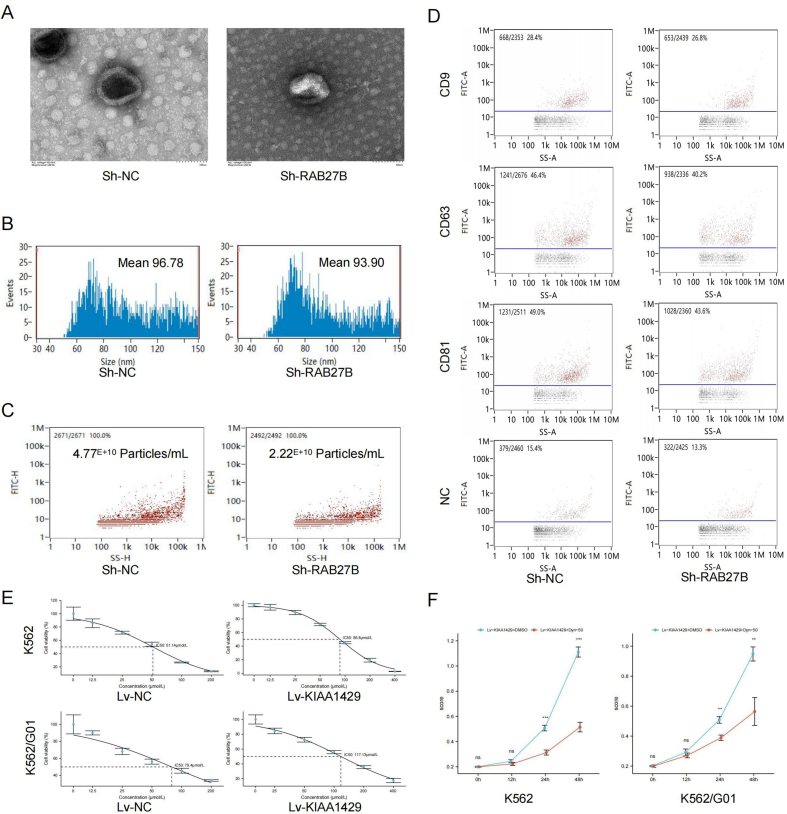
Exosome identification and Rab GTP inhibitor experiment. **(A)** An electron microscopic view of exosomes. **(B)** An exosome particle size distribution map. **(C)** Flow cytometry was used to detect the concentration of exosomes. **(D)** Nano-flow cytometry was used to detect the expression of exosome surface proteins. **(E)** The IC50 of dynamin was detected in CML cells by a cytotoxicity assay. **(F)** CCK-8 assays were used to assess the viability of CML cells after being treated with dynamin. ∗*P <* 0.05, ∗∗*P <* 0.01, ∗∗∗*P* < 0.001.

To verify whether the drug resistance of CML cells was associated with RAB27B-mediated drug transport, K562/G01 cells transfected with Sh-NC or sh-RAB27B were treated with 2 μmol/L imatinib for 48 h, and then cells were incubated in culture medium with no imatinib for another 24 h. Afterward, LC-MS was used to measure the imatinib concentration in the cells and culture medium. As can be seen in Figure 5I, the level of imatinib in the culture medium was lower, whereas its level inside the cells in the Sh-RNA27B group was higher than that observed in the Sh-NC group. The RAB27B knockdown also reduced the number of exosomes in the culture medium (Fig. S3A–D). These findings indicate that RAB27B knockdown inhibits the efflux of imatinib from cells, leading to drug enrichment inside the cells, which may also be associated with reduced exosome secretion. Of interest, we found that a Rab GTPase inhibitor, dynamin, also blocked the activity of KIAA1429 in CML cells (Fig. S3E, F).

### Inhibiting the KIAA1429 axis reduces the tumorigenic capacity of CML cell lines *in vivo*

The K562 parental cells, as well as K562 cells with KIAA1429, RAB27B, or YTHDF1 knockdown, were inoculated into BALB/C-nu mice to generate xenograft tumors. The results showed that the sizes of the tumors in the nude mice inoculated with the knockdown cells were smaller than those in the nude mice implanted with the parental K562 cells (Fig. 5J). Additionally, the tumor growth rate in the tumors with knockdown of any of these three targets was slower than that of the parental cells (Fig. 5K), indicating that knockdown of KIAA1429, RAB27B, or YTHDF1 significantly reduced the tumorigenic capacity of K562 cells. The body weight curve of the nude mice was also used to assess their overall nutritional status. We found that the body weight of the nude mice in the KIAA1429, RAB27B, and YTHDF1 knockdown K562 groups remained stable (Fig. 5L), and all had weights that were significantly higher than that in the K562 parental group.

### Rucaparib inhibited the KIAA1429 expression and reduced the proliferation and drug resistance of CML cells

**Figure 6:**
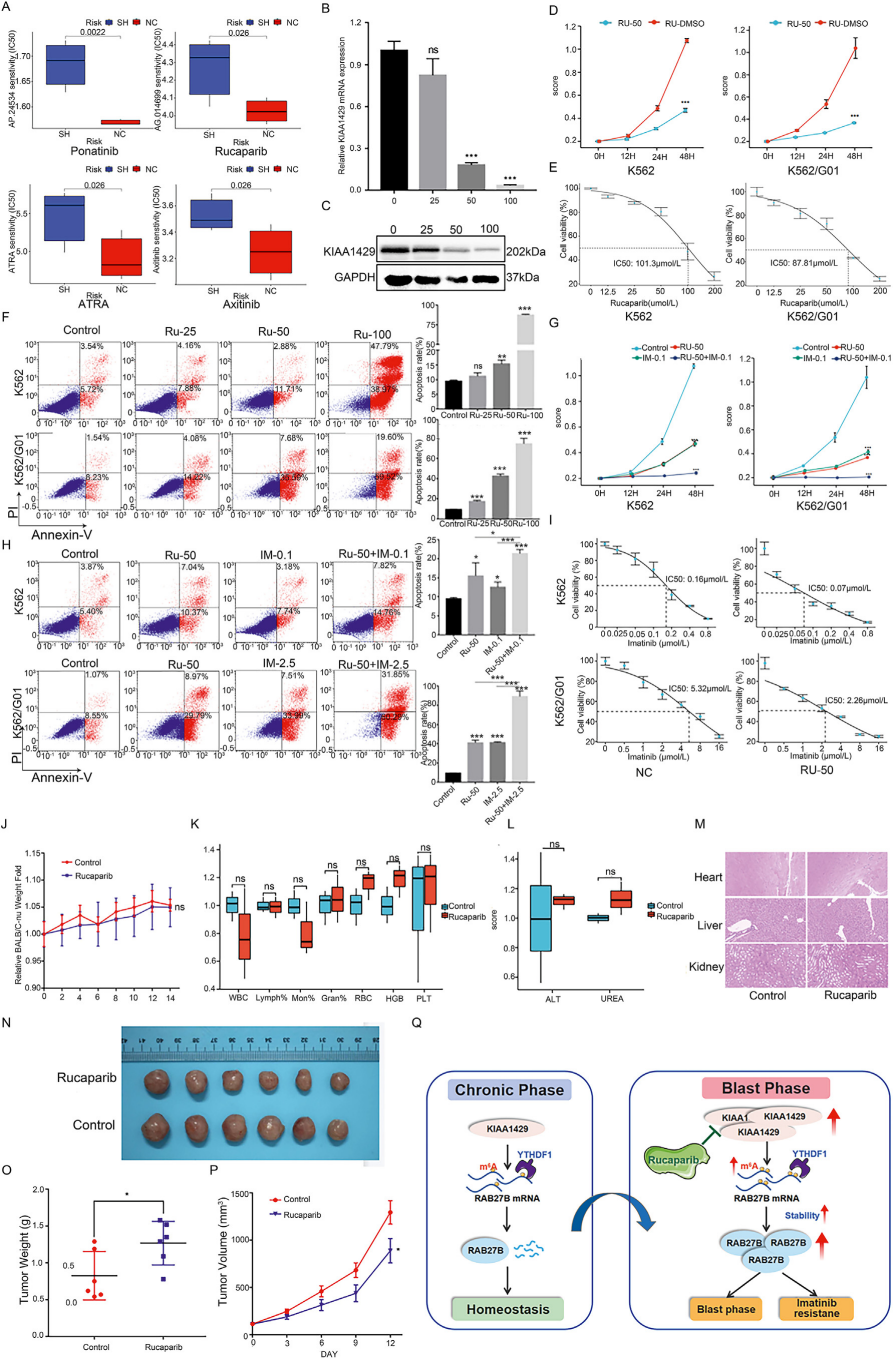
Rucaparib inhibited KIAA1429 expression and reduced the proliferation and drug resistance of CML *in vitro* and *in vivo*. **(A)** The Genomics of Drug Sensitivity in Cancer (GDSC) database and mRNA-seq data were analyzed comprehensively, and IC_50_ values were used to assess the sensitivity of the cells in each group to chemotherapeutic drugs. **(B)** RT-qPCR was used to detect the KIAA1429 mRNA expression after K562/G01 cells were treated with a concentration gradient of rucaparib. **(C)** Western blotting was used to detect the KIAA1429 protein expression after K562/G01 cells were treated with a concentration gradient of rucaparib. **(D)** CCK-8 assays were used to detect the viability of CML cells after treatment with rucaparib. **(E)** The IC_50_ of rucaparib was detected in CML cells by a cytotoxicity assay. **(F)** Flow cytometry was used to detect the apoptosis of CML cells after they were treated with a concentration gradient of rucaparib. **(G)** CCK-8 assays were used to detect the viability of CML cells after treatment with rucaparib alone or in combination with imatinib. **(H)** Flow cytometry was used to detect the apoptosis of CML cells after treatment with rucaparib alone or in combination with imatinib. **(I)** The IC_50_ of imatinib was detected by a cytotoxicity assay in CML cells after they were treated with rucaparib. **(J)** The body weight gain curves of BALB/C-nu mice after the intragastric administration of rucaparib. **(K)** Peripheral blood cell analysis of BALB/C-nu mice after the intragastric administration of rucaparib. **(L)** Biochemical analysis of the peripheral blood of BALB/C-nu mice after the intragastric administration of rucaparib. **(M)** HE staining of heart, liver, and kidney tissues excised from BALB/C-nu mice after the intragastric administration of rucaparib. **(N)** Images of tumors derived from BALB/C-nu mice in the control group and rucaparib group. **(O)** The tumor weights of BALB/C-nu mice in the control group and rucaparib group. **(P)** The tumor volume growth curve of BALB/C-nu mice in the control and rucaparib groups. **(Q)** KIAA1429 is highly expressed in BP-phase CML, which could enhance the stability of RAB27B mRNA to up-regulate RAB27B expression through the KIAA1429/m6A/YTHDF1 axis, consequently promoting CML progression. Rucaparib has the potential to inhibit the expression of KIAA1429, resulting in reduced CML progression. ^∗^*P* < 0.05, ^∗∗^*P* < 0.01, ^∗∗∗^*P* < 0.001.

**Fig. S4:**
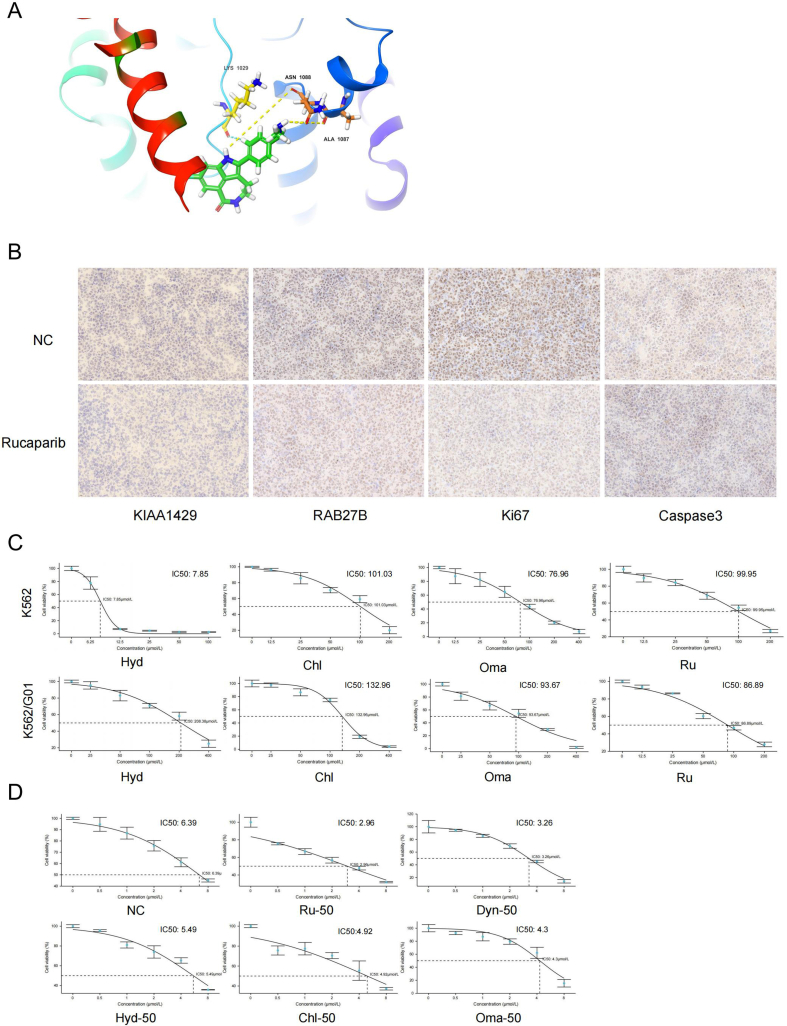
The effects and molecular mechanisms by which rucaparib exerts effects *in vitro* and *in vivo*. **(A)**The mechanism underlying the molecular docking rucaparib and KIAA11429. **(B)** Immunohistochemical staining of the frozen tumor tissues from the animal experiment. **(C)** IC50 determination of hydroxyurea, chlorambucil, omacetaxine, and rucaparib in K562 and K562/G01 cells. **(D)** IC50 determination of hydroxyurea, chlorambucil, omacetaxine, dynamin, and rucaparib in combination with 50 mmol/L imatinib in K562/G01 cells overexpressing KIAA1429.

The above studies revealed that KIAA1429 promotes the progression of CML. We, therefore, wanted to determine whether it might be possible to treat CML by targeting this gene. To screen for drugs that might specifically affect cells with KIAA1429 overexpression, the Genomics of Drug Sensitivity in Cancer (GDSC) database and mRNA-seq data were analyzed. After identifying drugs of interest, IC_50_ values were determined to assess the sensitivity of the cells in each group (parental and knockdown cells) to different chemotherapeutic drugs. The results obtained (Fig. 6A) showed that the KIAA1429 knockdown group had higher IC_50_ values for ponatinib (*P* = 0.002), rucaparib (*P* = 0.026), axitinib (*P* = 0.026), and ATRA (*P* = 0.026) than the parental cells. These findings indicated that cells with KIAA1429 overexpression may be more sensitive to these anti-tumor drugs. Ponatinib, axitinib, and ATRA have already been applied for CML treatment in clinical practice. Thus, in this study, we focused on the possible benefits of treatment with rucaparib. Molecular docking studies using the AutoDockTools-1.5.6 software were performed to determine whether there was an interaction between rucaparib and KIAA11429 at the molecular level (Fig. S4A). The docking position was set in a cube in the center of the initial ligand. The lower the docking fraction (the greater the negative value), the higher the binding force between the compound and the protein. The molecular docking results showed that the docking fraction of rucaparib to KIAA1429 protein was −4.501 kJ/mol and the key amino acids at the binding site were LYS1029, ASN1088, and ALA1087, indicating that rucaparib had a strong interaction with the KIAA1429 protein.

Interestingly, a 48-h treatment with concentration gradients of rucaparib decreased the cell expression of KIAA1429 at both the mRNA and protein levels (Fig. 6B, C). The degrees of this reduction were positively associated with the drug concentrations, indicating that rucaparib could inhibit the expression of KIAA1429. In addition, rucaparib suppressed the proliferation of CML cells (Fig. 6D). The IC_50_ values in the K562 and K562/G01 cells were 101.3 μM and 87.81 μM (Fig. 6E), respectively. The 48-h rucaparib treatment also dose-dependently promoted the apoptosis of CML cells (Fig. 6F). These findings demonstrated that rucaparib suppressed the proliferation and promoted the apoptosis of CML cells. These effects were more pronounced in imatinib-resistant CML cells than in sensitive cells.

Based on the above observations, we further investigated whether rucaparib could enhance the response of drug-resistant CML cells to imatinib. Low concentrations of rucaparib and imatinib (at approximately 50% of the IC_50_ value for each) were used to treat cells independently or in combination, and then the proliferation and apoptosis of cells were observed. We found that the proliferation of cells in the rucaparib + imatinib group was significantly lower than that in the control, rucaparib, and imatinib groups (Fig. 6G). The apoptosis rate in the rucaparib + imatinib group was also significantly higher than that in the other three groups (Fig. 6H). Of note, rucaparib treatment significantly decreased the IC_50_ of imatinib (Fig. 6I).

We next investigated whether rucaparib could be used to treat CML *in vivo*. First, a quick 1-week toxicity study was performed in BALB/C-nu mice to confirm the safety of the treatment. The body weight (Fig. 6J), routine blood parameters (Fig. 6K), and biochemical indicators such as ALT and urea (Fig. 6L) in the treatment group were not significantly different from those in the control group, and no tissue injury or inflammatory lesions were found in the treatment group (Fig. 6M). These findings indicated that this dose of rucaparib exerted no overt toxicity *in vivo*, and could be used for further studies.

The nude mice subcutaneously injected with CML cells were treated with rucaparib to assess its therapeutic effects. The tumor size (Fig. 6N), weight (Fig. 6O), and growth (Fig. 6P) in the treatment group were significantly lower than those in the control group, and the expression levels of KIAA1429, RAB27B, and Ki67 in the tumor tissues in the treatment group were significantly lower than those in the normal saline control group, while the expression of caspase-3 was significantly higher than that in the control group (Fig. S4B), indicating that rucaparib inhibited tumorigenesis in the CML cells *in vivo*, suggesting that it may have therapeutic value.

## Discussion

The progression to blast phase CML and the resistance of CML to TKIs are current challenges in clinical practice^3^^,^^6^ Previous studies have shown that m^6^A modification plays an important role in the development and progression of human diseases.^26^^,^^27^ KIAA1429 is highly expressed in various tumors and is closely associated with tumor progression and the poor prognosis of patients.^28^ In this study, we first established that both the expression of KIAA1429 and the total m^6^A modification level were significantly higher in the BP phase of CML than in the CP phase. In addition, the KIAA1429 expression was also more up-regulated in imatinib-resistant K562/G01 cells than in the imatinib-sensitive K562 cells. These results suggest that KIAA1429 is involved in the malignant progression and imatinib resistance of CML cells. We are also the first to demonstrate that KIAA1429 regulates the total m^6^A modification levels in CML cells.

In agreement with our findings, Schwartz et al^29^ previously reported that KIAA1429 knockdown resulted in a four-fold decrease in the peak m^6^A score, which was more prominent in human cells after METTL3 and METTL14 knockdown. The cell function experiments and *in vivo* experiments in the present study indicated that KIAA1429 could promote the malignant biological behaviors of CML, enhancing disease development and progression. Previous research has revealed that RAB27B influences cell signal transduction and is closely associated with exosome release.^30^, ^31^, ^32^ Hence, it plays an important role in the maturation and secretion of zymogen granules^33^^,^^34^ and, therefore, could be used as a tumor marker for disease analysis and prognostic prediction.^35^^,^^36^ In this study, we established that RAB27B expression also plays a similar role in CML. The investigation of its underlying mechanisms showed that the processes mediated by KIAA1429 were at least partly dependent on RAB27B.

We, therefore, performed a more detailed examination of the function of RAB27B in CML cells. Knocking down RAB27B expression inhibited the proliferation and imatinib resistance of CML cells, and promoted CML cell apoptosis, suggesting that RAB27B promotes the development and/or progression of CML. We identified three m^6^A modification sites, two of which are located in the 3′UTR region, and are related to mRNA stability. The other site is located in the CDS sequence. Thus, in addition to its impact on mRNA stability, the m^6^A modification may also affect its translation efficiency. The m^6^A modification ultimately produces an effect through the m^6^A reader protein,^37^, ^38^, ^39^ and we elucidated that YTHDF1 is the reader protein for RAB27B. More specifically, inhibiting KIAA1429 expression could reduce the m^6^A modification of RAB27B mRNA, which is then recognized by YTHDF1, resulting in reduced stability of the mRNA, decreased expression of RAB27B, and consequently reduced proliferation and drug resistance of CML cells.

The progression, response to treatment, and overall clinical outcomes of CML patients are also influenced by the accumulation of other abnormal genetic and epigenetic factors, not only the Bcr/Abl1 oncoprotein.^5^^,^^40^^,^^41^ Thus, both genetic and epigenetic factors could influence the efficacy of CML treatment. Notably, novel targets (such as the m^6^A modification) may play an active role in tumor progression and drug resistance.^27^^,^^42^^,^^43^ Previously, several inhibitors of m^6^A regulatory factors have been developed as anti-cancer drugs, with promising results. For instance, STM2457 targets METTL3 and METTLl4, the key components of m^6^A methyltransferase, and were reported to significantly inhibit the proliferation, induce the differentiation, and increase the apoptosis of leukemia cells.^19^^,^^44^ Notably, STM2457 exerts no significant effects on normal hematopoietic stem cells or other normal cells. Based on this paradigm, we wanted to establish an approach targeting KIAA1429 expression to provide a more effective treatment for CML. The bioinformatics analysis in this study identified four candidate drugs (ponatinib, rucaparib, axitinib, and ATRA) out of 138 anti-tumor drugs. Ponatinib and axitinib are second-generation TKIs that effectively inhibit the Bcr/Abl1 signaling in CML cells with the T315I^+^ complex mutation.^45^, ^46^, ^47^ ATRA reduces DNA damage repair and inhibits the acquisition of the Bcr/Abl1 mutation during the treatment of CML, consequently reducing the adaptive drug resistance in CML.^48^^,^^49^ Recent studies have established that a PARP inhibitor could be used alone or in combination with cytotoxic drugs, hypomethylation agents, or molecularly targeted drugs for the treatment of acute leukemia.^50^^,^^51^ Nevertheless, the use of PARP inhibitors such as rucaparib in the treatment of CML has not been reported.

In this study, *in vitro* examinations showed that rucaparib could inhibit the proliferation and promote the apoptosis of CML cells. Furthermore, it increased the sensitivity of CML cells to imatinib. Interestingly the IC_50_ of rucaparib in K562/G01 cells was lower than that in K562 cells, indicating that rucaparib has high treatment potential for CML-BC or drug-resistant CML. In addition, rucaparib exerted more potent effects than hydroxyurea, chlorambucil, and omacetaxine, three chemotherapeutic drugs approved for the treatment of CML (Fig. S4C, D). We performed additional *in vivo* experiments on animals to verify our findings. The results of these studies showed that rucaparib effectively inhibited tumor growth without any overt toxicity to the mice, and thus has a high potential for clinical application.

However, we also noticed that the effects of m^6^A modification on drug responses are not consistent or easy to understand. Therefore, more experiments are needed to explore the therapeutic effects of the drug alone and in combination with other approaches. It is also reasonable to anticipate the development and discovery of more m^6^A-related protein inhibitors that may be useful for the treatment of CML, and the custom design of novel inhibitors should be considered.

## Conclusions

KIAA1429 is highly expressed in CML-BC and could enhance the RAB27B mRNA stability to up-regulate RAB27B expression through the KIAA1429/m^6^A/YTHDF1 axis, consequently promoting CML progression. Rucaparib has the potential to inhibit the expression of KIAA1429, resulting in reduced progression of CML. This study provides theoretical evidence that sheds light on the mechanisms underlying CML progression and drug resistance, which may have implications for the clinical treatment of CML. Additionally, it provides novel insights into a potential option for the treatment of imatinib-resistant CML (Fig. 6Q).

## Ethics declaration

All animal experiments were in accordance with the National Institutes of Health guide for the care and use of laboratory animals (NIH Publications No. 8023, revised 1978) and were conducted with the approval of the Ethics Committee of Nanchang University, Jiangxi, China. This study had approval from the Ethics Committee of the Second Affiliated Hospital of Nanchang University, Jiangxi, China; all participants provided signed informed consent. The protocols complied with the Declaration of Helsinki.

## Author contributions

Conceptualization: Fangyi Yao, Bo Huang, and Xiaozhong Wang; Data curation: Fangyi Yao; Formal analysis: Fangmin Zhong and Junyao Jiang; Funding acquisition: Xiaozhong Wang; Investigation: Ying Cheng and Yanmei Xu; Methodology: Fangyi Yao and Fangmin Zhong; Resources: Shuai Xu and Jing Liu; Software: Fangyi Yao and Fangmin Zhong; Supervision: Xiaozhong Wang; Validation: Jin Lin and Jing Zhang; Visualization: Shuqi Li and Meiyong Li; Writing-original draft: Fangyi Yao; Writing-review &editing: Bo Huang and Xiaozhong Wang. The authors read and approved the final manuscript.

## Conflict of interests

The authors declare no conflict of interests.

## Funding

This work was supported by grants from the 10.13039/501100001809National Natural Science Foundation of China (No. 81860034, 82160405, 82160038, 82260035) and the Natural Science Foundations of Jiangxi Province, China (No. 20224BAB216037, 20212ACB206016).
