# Supplementary material for: The m6A regulator KIAA1429 stabilizes RAB27B mRNA and promotes the progression of chronic myeloid leukemia and resistance to targeted therapy
Source: Genes Dis. 2023 Apr 12;11(2):993–1008. doi: 10.1016/j.gendis.2023.03.016 (PMC10491918; doi:10.1016/j.gendis.2023.03.016)
Supplement: Multimedia component 1 [file mmc1.docx]

Table S1 Target sequence

| Gene symbol | Target sequence |
| --- | --- |
| Sh1-KIAA1429  sense strands | 5’-CCGGCGGAATATGAAGCAACAAATTCTCGAGAATTTGTTGCTTCATATTCCGTTTTTG- 3’ |
| Sh1-KIAA1429  antisense strands | 5’-AATTCAAAAACGGAATATGAAGCAACAAATTCTCGAGAATTTGTTGCTTCATATTCCG- 3’ |
| Sh2-KIAA1429  sense strands | 5’-CCGGCGCTGAGCAAAGTTCTCATATCTCGAGATATGAGAACTTTGCTCAGCGTTTTTG- 3’ |
| Sh2-KIAA1429  antisense strands | 5’-AATTCAAAAACGCTGAGCAAAGTTCTCATATCTCGAGATATGAGAACTTTGCTCAGCG- 3’ |
| Sh-RAB27B  sense strands | 5’-GATCCGCCAGTCAACAGAGCTTCTTAATTCAAGAGATTAAGAAGCTCTGTTGACTGGTTTTTTG- 3 |
| Sh-RAB27B  antisense strands | 5’-AATTCAAAAAACCAGTCAACAGAGCTTCTTAATCTCTTGAATTAAGAAGCTCTGTTGACTGGCG- 3’ |
| Sh-YTHDF1  sense strands | 5’-CCGGCCCGAAAGAGTTTGAGTGGAACTCGAGTTCCACTCAAACTCTTTCGGGTTTTTG- 3’ |
| Sh-YTHDF1  antisense strands | 5’-AATTCAAAAACCCGAAAGAGTTTGAGTGGAACTCGAGTTCCACTCAAACTCTTTCGGG- 3 |
