# Supplementary material for: The m6A regulator KIAA1429 stabilizes RAB27B mRNA and promotes the progression of chronic myeloid leukemia and resistance to targeted therapy
Source: Genes Dis. 2023 Apr 12;11(2):993–1008. doi: 10.1016/j.gendis.2023.03.016 (PMC10491918; doi:10.1016/j.gendis.2023.03.016)
Supplement: Multimedia component 2 [file mmc2.docx]

Table S2 Primers RT-qPCR.

| Gene | Forward primer(5'-3') | Reverse primer(5'-3') |
| --- | --- | --- |
| KIAA1429 | CTTGGCAAGTGGCTTGAACC | ACGTAAGGCAGTGGTAAGGC |
| RAB27B | AAGGCAGACCTACCAGATCAGAG | TTCTCCACACACTGTTCCATTCG |
| YTHDF1 | TAAGGAAATCCAATGGACGG | TTTGAGCCCTACCTTACTGGA |
| GAPDH | TGCCAAATATGATGACATCAAGAA | GGAGTGGGTGTCGCTGTTG |
