# Supplementary material for: The m6A regulator KIAA1429 stabilizes RAB27B mRNA and promotes the progression of chronic myeloid leukemia and resistance to targeted therapy
Source: Genes Dis. 2023 Apr 12;11(2):993–1008. doi: 10.1016/j.gendis.2023.03.016 (PMC10491918; doi:10.1016/j.gendis.2023.03.016)
Supplement: Multimedia component 3 [file mmc3.docx]

**Table S3 Intersection genes mRNA-seq information.**

| **Geneid** | **Chr** | **Length** | **Sh1-**  **KIAA1429-1** | **Sh1-**  **KIAA1429-2** | **Sh1-**  **KIAA1429-3** | **Sh-NC-1** | **Sh-NC-2** | **Sh-NC-3** | **PValue** | **FDR** | **logFC** |
| --- | --- | --- | --- | --- | --- | --- | --- | --- | --- | --- | --- |
| ABCG2 | 4 | 4848 | 1.897 | 5.483 | 4.746 | 0.757 | 1.753 | 2.094 | 0.012 | 0.332 | 1.549 |
| ANKRD33B | 5 | 9783 | 0.314 | 0.166 | 0.478 | 0.904 | 0.990 | 0.616 | 0.016 | 0.332 | -1.237 |
| APOL1 | 22 | 3519 | 49.600 | 94.065 | 54.151 | 27.095 | 28.962 | 32.248 | 0.003 | 0.274 | 1.346 |
| APOL2 | 22 | 3484 | 12.341 | 21.321 | 13.166 | 5.925 | 5.640 | 7.644 | 0.002 | 0.241 | 1.465 |
| ARL2 | 11 | 2084 | 34.144 | 38.352 | 19.086 | 17.357 | 14.200 | 15.646 | 0.012 | 0.332 | 1.162 |
| ATP6V0E2 | 7 | 3335 | 4.313 | 3.523 | 2.290 | 2.643 | 1.503 | 1.392 | 0.034 | 0.375 | 1.074 |
| BCAM | 19 | 5024 | 5.994 | 13.975 | 4.044 | 1.671 | 2.873 | 1.075 | 0.003 | 0.273 | 2.314 |
| BCAS1 | 20 | 8186 | 0.070 | 0.075 | 0.051 | 0.173 | 0.461 | 0.113 | 0.024 | 0.351 | -1.714 |
| BCL6 | 3 | 5938 | 0.712 | 2.620 | 2.168 | 0.459 | 0.679 | 0.503 | 0.003 | 0.273 | 1.896 |
| C10orf54 | 10 | 5128 | 0.162 | 0.159 | 0.315 | 0.849 | 0.652 | 0.656 | 0.005 | 0.293 | -1.618 |
| CADM4 | 19 | 2246 | 3.138 | 4.765 | 2.463 | 1.892 | 2.245 | 1.015 | 0.022 | 0.340 | 1.211 |
| CARHSP1 | 16 | 5577 | 10.869 | 17.173 | 7.268 | 4.769 | 5.601 | 5.248 | 0.006 | 0.304 | 1.382 |
| CCDC80 | 3 | 13071 | 0.025 | 0.107 | 0.135 | 0.273 | 0.328 | 0.166 | 0.041 | 0.390 | -1.390 |
| CD163L1 | 12 | 5690 | 0.090 | 0.286 | 0.267 | 0.092 | 0.086 | 0.052 | 0.023 | 0.348 | 1.619 |
| CDCA7 | 2 | 3934 | 5.684 | 3.467 | 13.247 | 19.789 | 18.707 | 22.677 | 0.013 | 0.332 | -1.336 |
| CDK19 | 6 | 7492 | 0.325 | 0.330 | 0.657 | 1.491 | 3.054 | 1.178 | 0.002 | 0.234 | -1.970 |
| CERS4 | 19 | 4059 | 1.681 | 2.264 | 1.387 | 1.002 | 1.257 | 0.642 | 0.029 | 0.366 | 1.078 |
| CHML | 1 | 7661 | 2.559 | 2.874 | 7.879 | 8.549 | 10.095 | 13.310 | 0.021 | 0.339 | -1.155 |
| CHRD | 3 | 6620 | 1.099 | 1.063 | 0.462 | 0.594 | 0.387 | 0.267 | 0.033 | 0.373 | 1.286 |
| CHST3 | 10 | 6970 | 6.412 | 6.864 | 6.469 | 3.071 | 3.274 | 2.773 | 0.002 | 0.251 | 1.296 |
| CMTM1 | 16 | 1465 | 0.066 | 0.079 | 0.501 | 0.412 | 1.082 | 1.093 | 0.046 | 0.399 | -1.867 |
| CNPY2 | 12 | 2956 | 61.333 | 82.492 | 44.144 | 34.758 | 32.021 | 36.085 | 0.012 | 0.332 | 1.067 |
| COL6A3 | 2 | 19633 | 0.000 | 0.003 | 0.007 | 0.102 | 0.088 | 0.030 | 0.002 | 0.223 | -4.135 |
| COL6A5 | 3 | 9586 | 6.009 | 8.978 | 8.278 | 3.861 | 2.874 | 5.254 | 0.007 | 0.311 | 1.115 |
| CSNK1G3 | 5 | 5000 | 0.916 | 0.989 | 3.079 | 3.258 | 4.247 | 3.730 | 0.038 | 0.379 | -1.064 |
| CTSF | 11 | 2896 | 26.329 | 50.125 | 11.309 | 6.539 | 9.273 | 3.844 | 0.003 | 0.273 | 2.388 |
| CXCL8 | 4 | 2274 | 0.056 | 0.026 | 0.280 | 0.715 | 0.482 | 0.358 | 0.036 | 0.375 | -1.971 |
| DBP | 19 | 6544 | 0.494 | 1.542 | 0.307 | 0.301 | 0.282 | 0.203 | 0.021 | 0.339 | 1.783 |
| DDX60L | 4 | 12156 | 0.092 | 0.038 | 0.127 | 0.192 | 0.410 | 0.228 | 0.018 | 0.332 | -1.522 |
| DMTN | 8 | 4473 | 12.456 | 31.783 | 11.322 | 4.076 | 9.978 | 4.279 | 0.008 | 0.312 | 1.812 |
| DOCK2 | 5 | 12890 | 0.005 | 0.036 | 0.023 | 0.106 | 0.232 | 0.177 | 0.003 | 0.270 | -2.824 |
| DSE | 6 | 15016 | 0.604 | 0.771 | 2.272 | 2.528 | 3.335 | 2.952 | 0.026 | 0.357 | -1.169 |
| EDEM2 | 20 | 1919 | 8.548 | 16.202 | 8.380 | 5.427 | 4.520 | 5.206 | 0.006 | 0.305 | 1.313 |
| ENC1 | 5 | 5858 | 0.022 | 0.040 | 0.217 | 0.690 | 0.920 | 0.487 | 0.005 | 0.293 | -2.793 |
| ESPN | 1 | 6943 | 40.690 | 71.223 | 15.466 | 6.067 | 7.914 | 4.203 | 0.001 | 0.206 | 3.038 |
| ETV5 | 3 | 5802 | 0.707 | 0.391 | 1.160 | 1.980 | 2.379 | 0.992 | 0.042 | 0.392 | -1.095 |
| FAM89A | 1 | 2394 | 1.807 | 1.604 | 1.155 | 3.846 | 4.983 | 2.404 | 0.026 | 0.357 | -1.090 |
| FAM9B | X | 6843 | 0.009 | 0.017 | 0.000 | 0.061 | 0.387 | 0.159 | 0.004 | 0.283 | -4.286 |
| FBXO2 | 1 | 2333 | 1.593 | 3.453 | 1.311 | 0.675 | 1.223 | 0.349 | 0.017 | 0.332 | 1.712 |
| FTH1 | 11 | 2030 | 1514.565 | 2030.076 | 763.545 | 557.338 | 707.799 | 507.652 | 0.008 | 0.311 | 1.500 |
| FTH1P8 | X | 514 | 18.076 | 26.991 | 6.096 | 4.389 | 5.550 | 5.599 | 0.008 | 0.311 | 1.944 |
| FTL | 19 | 878 | 519.318 | 537.771 | 229.833 | 247.844 | 223.919 | 166.571 | 0.018 | 0.332 | 1.231 |
| GAS6 | 13 | 6006 | 4.614 | 8.828 | 2.629 | 1.625 | 1.761 | 0.958 | 0.003 | 0.269 | 2.102 |
| GATS | 7 | 4860 | 1.398 | 2.561 | 2.080 | 0.880 | 1.150 | 1.184 | 0.024 | 0.351 | 1.078 |
| GNAL | 18 | 8445 | 1.373 | 4.460 | 2.681 | 0.301 | 0.440 | 0.527 | 0.000 | 0.190 | 2.912 |
| GPX8 | 5 | 3868 | 0.315 | 0.286 | 0.203 | 1.085 | 2.339 | 0.576 | 0.005 | 0.293 | -2.099 |
| HDAC11 | 3 | 5281 | 2.766 | 5.370 | 2.424 | 0.869 | 1.233 | 0.797 | 0.001 | 0.218 | 2.065 |
| HDAC9 | 7 | 16827 | 0.027 | 0.035 | 0.164 | 0.156 | 0.276 | 0.310 | 0.036 | 0.375 | -1.620 |
| HIGD2A | 5 | 668 | 23.645 | 20.290 | 10.773 | 8.599 | 10.266 | 11.949 | 0.032 | 0.372 | 1.044 |
| HOXB7 | 17 | 1528 | 63.572 | 76.690 | 37.998 | 29.715 | 28.833 | 31.040 | 0.009 | 0.315 | 1.198 |
| HSD3B7 | 16 | 2495 | 3.724 | 6.598 | 3.512 | 2.229 | 3.546 | 1.980 | 0.028 | 0.366 | 1.034 |
| IDH3G | X | 2717 | 26.519 | 27.597 | 14.946 | 13.004 | 9.142 | 10.801 | 0.008 | 0.311 | 1.271 |
| IFI27 | 14 | 4147 | 0.371 | 1.136 | 0.236 | 0.145 | 0.236 | 0.065 | 0.018 | 0.332 | 2.177 |
| IGFBP4 | 17 | 2200 | 1.223 | 0.912 | 0.779 | 5.294 | 10.059 | 1.654 | 0.005 | 0.293 | -2.326 |
| IL16 | 15 | 12094 | 0.967 | 0.659 | 0.678 | 2.269 | 1.611 | 1.661 | 0.016 | 0.332 | -1.075 |
| INHBE | 12 | 3015 | 1.392 | 0.521 | 0.520 | 3.506 | 10.044 | 0.738 | 0.027 | 0.358 | -2.303 |
| INPP5J | 22 | 4822 | 1.415 | 3.529 | 2.482 | 0.729 | 0.980 | 1.070 | 0.003 | 0.273 | 1.584 |
| KCTD11 | 17 | 3056 | 3.177 | 3.674 | 1.129 | 1.416 | 1.263 | 0.915 | 0.026 | 0.357 | 1.374 |
| KIAA0513 | 16 | 8837 | 4.097 | 6.194 | 4.490 | 2.386 | 2.576 | 1.687 | 0.002 | 0.248 | 1.336 |
| KIAA1429 | 8 | 10771 | 1.374 | 1.680 | 8.577 | 8.226 | 12.292 | 13.187 | 0.041 | 0.391 | -1.464 |
| KLHDC8B | 3 | 2158 | 23.412 | 33.101 | 13.464 | 8.095 | 13.366 | 5.787 | 0.007 | 0.311 | 1.582 |
| KYNU | 2 | 17600 | 0.015 | 0.069 | 0.072 | 0.086 | 0.242 | 0.210 | 0.030 | 0.367 | -1.633 |
| L3MBTL1 | 20 | 10674 | 0.300 | 0.401 | 0.495 | 0.885 | 1.298 | 0.547 | 0.034 | 0.375 | -1.024 |
| LMOD1 | 1 | 3971 | 3.106 | 8.994 | 6.139 | 2.708 | 3.766 | 1.381 | 0.018 | 0.332 | 1.389 |
| MICA | 6 | 2386 | 19.859 | 29.146 | 17.317 | 10.554 | 16.182 | 8.033 | 0.013 | 0.332 | 1.133 |
| MICALCL | 11 | 3448 | 0.232 | 0.152 | 0.639 | 0.609 | 1.137 | 1.126 | 0.036 | 0.375 | -1.363 |
| MILR1 | 17 | 1816 | 0.141 | 0.336 | 0.391 | 0.722 | 0.920 | 1.091 | 0.021 | 0.337 | -1.500 |
| MIR1199 | 19 | 2354 | 2.477 | 2.521 | 1.206 | 1.270 | 0.600 | 0.669 | 0.017 | 0.332 | 1.490 |
| MLLT11 | 1 | 4075 | 2.311 | 1.128 | 4.103 | 8.407 | 7.615 | 4.437 | 0.019 | 0.333 | -1.304 |
| MME | 3 | 9620 | 0.100 | 0.193 | 0.290 | 0.387 | 1.249 | 0.494 | 0.011 | 0.332 | -1.711 |
| MRAP2 | 6 | 2153 | 5.878 | 11.456 | 11.835 | 4.264 | 4.430 | 6.280 | 0.012 | 0.332 | 1.109 |
| MYB | 6 | 5493 | 4.648 | 2.076 | 17.281 | 39.869 | 32.534 | 44.704 | 0.008 | 0.311 | -2.210 |
| NEXN | 1 | 4436 | 1.257 | 1.154 | 2.185 | 3.577 | 7.418 | 2.118 | 0.018 | 0.332 | -1.349 |
| NR6A1 | 9 | 7124 | 1.223 | 0.853 | 3.292 | 4.334 | 4.000 | 4.043 | 0.034 | 0.375 | -1.096 |
| PECAM1 | 17 | 7759 | 0.025 | 0.052 | 0.000 | 0.264 | 0.249 | 0.098 | 0.025 | 0.354 | -2.763 |
| PHACTR2 | 6 | 11224 | 0.225 | 0.254 | 0.783 | 0.893 | 1.017 | 1.217 | 0.027 | 0.360 | -1.202 |
| PHOSPHO1 | 17 | 2375 | 0.540 | 1.298 | 0.124 | 0.000 | 0.170 | 0.114 | 0.030 | 0.367 | 3.016 |
| PIGCP1 | 11 | 895 | 0.286 | 0.390 | 1.832 | 3.663 | 4.733 | 3.397 | 0.010 | 0.326 | -2.123 |
| PINK1 | 1 | 4836 | 6.519 | 12.293 | 5.254 | 3.423 | 5.666 | 2.290 | 0.021 | 0.337 | 1.290 |
| PLAGL1 | 6 | 8719 | 0.184 | 0.247 | 0.657 | 1.194 | 1.008 | 1.205 | 0.008 | 0.311 | -1.540 |
| PLAU | 10 | 2950 | 0.369 | 0.237 | 0.149 | 1.538 | 1.309 | 0.690 | 0.006 | 0.298 | -2.015 |
| PPP1R14B | 11 | 1655 | 182.393 | 132.929 | 79.512 | 72.352 | 48.593 | 72.325 | 0.016 | 0.332 | 1.243 |
| PROCR | 20 | 1696 | 13.544 | 41.518 | 33.628 | 8.042 | 10.483 | 10.981 | 0.002 | 0.251 | 1.740 |
| PRSS23 | 11 | 11725 | 0.036 | 0.032 | 0.317 | 0.333 | 0.868 | 0.678 | 0.025 | 0.356 | -2.203 |
| PTGS1 | 9 | 5790 | 2.623 | 7.610 | 10.104 | 1.558 | 2.334 | 1.613 | 0.002 | 0.223 | 2.015 |
| PTK2B | 8 | 7933 | 7.746 | 11.685 | 6.301 | 3.799 | 3.974 | 2.382 | 0.002 | 0.262 | 1.540 |
| PTP4A3 | 8 | 2877 | 35.657 | 24.606 | 17.059 | 11.460 | 6.320 | 8.974 | 0.003 | 0.268 | 1.738 |
| PTPRC | 1 | 8371 | 1.171 | 1.445 | 5.015 | 5.349 | 6.427 | 7.106 | 0.033 | 0.375 | -1.215 |
| QRICH2 | 17 | 5775 | 1.043 | 0.972 | 0.899 | 0.645 | 0.190 | 0.527 | 0.030 | 0.367 | 1.266 |
| R3HCC1 | 8 | 2630 | 31.465 | 25.336 | 17.320 | 16.387 | 9.959 | 15.133 | 0.023 | 0.347 | 1.039 |
| RAB27B | 18 | 7432 | 0.026 | 0.023 | 0.013 | 0.289 | 0.248 | 0.066 | 0.003 | 0.271 | -3.065 |
| RAB3D | 19 | 4417 | 4.751 | 5.255 | 3.536 | 2.631 | 2.766 | 1.752 | 0.012 | 0.332 | 1.121 |
| RAB6B | 3 | 6005 | 5.714 | 8.428 | 5.148 | 3.328 | 5.470 | 2.016 | 0.032 | 0.373 | 1.038 |
| RAD51AP1 | 12 | 2558 | 4.121 | 6.288 | 10.488 | 10.787 | 25.435 | 11.218 | 0.036 | 0.375 | -1.036 |
| RARRES3 | 11 | 1299 | 2.614 | 11.531 | 2.299 | 0.606 | 1.287 | 0.543 | 0.003 | 0.273 | 2.968 |
| RASSF4 | 10 | 9481 | 0.014 | 0.117 | 0.147 | 0.033 | 0.030 | 0.011 | 0.044 | 0.397 | 1.994 |
| RPL26L1 | 5 | 1164 | 72.828 | 72.362 | 42.058 | 26.816 | 28.196 | 34.146 | 0.006 | 0.297 | 1.276 |
| RSAD2 | 2 | 4834 | 1.312 | 5.710 | 4.572 | 0.336 | 0.393 | 0.607 | 0.000 | 0.190 | 3.256 |
| S1PR3 | 9 | 8754 | 0.007 | 0.007 | 0.022 | 0.126 | 0.145 | 0.093 | 0.001 | 0.218 | -3.112 |
| SALL2 | 14 | 5382 | 1.786 | 1.800 | 4.453 | 6.955 | 6.061 | 5.332 | 0.016 | 0.332 | -1.070 |
| SERPINB6 | 6 | 4729 | 73.330 | 59.947 | 31.072 | 35.305 | 28.022 | 23.021 | 0.023 | 0.348 | 1.148 |
| SETD7 | 4 | 13916 | 0.592 | 0.510 | 1.993 | 2.239 | 3.512 | 1.926 | 0.037 | 0.376 | -1.202 |
| SKP2 | 5 | 3837 | 2.497 | 2.350 | 5.773 | 7.082 | 9.019 | 9.021 | 0.012 | 0.332 | -1.115 |
| SLC7A8 | 14 | 5991 | 3.219 | 5.068 | 3.040 | 2.465 | 1.837 | 1.323 | 0.011 | 0.330 | 1.196 |
| SLIT2 | 4 | 9712 | 0.020 | 0.006 | 0.005 | 0.154 | 0.145 | 0.106 | 0.001 | 0.218 | -3.517 |
| SNAI3 | 16 | 1732 | 1.258 | 2.334 | 0.650 | 0.318 | 0.383 | 0.376 | 0.005 | 0.293 | 2.183 |
| SNN | 16 | 3312 | 0.638 | 1.906 | 0.665 | 0.412 | 0.679 | 0.246 | 0.036 | 0.375 | 1.467 |
| SPRED2 | 2 | 5154 | 0.752 | 0.604 | 2.341 | 2.870 | 2.913 | 2.844 | 0.039 | 0.382 | -1.117 |
| SPRY4 | 5 | 5930 | 1.702 | 0.441 | 2.386 | 6.476 | 3.785 | 2.971 | 0.037 | 0.375 | -1.408 |
| STON1 | 2 | 7709 | 0.050 | 0.030 | 0.013 | 0.136 | 0.172 | 0.070 | 0.032 | 0.373 | -1.812 |
| STX8 | 17 | 3779 | 8.461 | 12.006 | 4.871 | 4.415 | 4.895 | 4.030 | 0.019 | 0.333 | 1.136 |
| STXBP5 | 6 | 10504 | 0.708 | 0.853 | 2.843 | 3.748 | 2.521 | 5.466 | 0.032 | 0.372 | -1.329 |
| TBCB | 19 | 3326 | 18.283 | 15.461 | 9.450 | 9.882 | 4.704 | 9.142 | 0.035 | 0.375 | 1.065 |
| TMEM156 | 4 | 2562 | 0.225 | 0.170 | 0.191 | 0.829 | 1.507 | 0.593 | 0.003 | 0.269 | -2.121 |
| TMEM55A | 8 | 3066 | 1.024 | 1.556 | 2.754 | 2.746 | 7.594 | 2.683 | 0.046 | 0.399 | -1.139 |
| TMEM63B | 6 | 4425 | 36.751 | 55.199 | 23.133 | 15.240 | 15.837 | 13.197 | 0.003 | 0.271 | 1.585 |
| TNFRSF21 | 6 | 3595 | 0.107 | 0.065 | 0.204 | 0.555 | 0.785 | 0.483 | 0.003 | 0.273 | -2.118 |
| TPM4 | 19 | 7414 | 9.334 | 14.637 | 25.788 | 34.435 | 59.389 | 24.413 | 0.026 | 0.358 | -1.111 |
| TSPYL1 | 6 | 3326 | 4.229 | 5.352 | 12.791 | 14.448 | 18.990 | 16.709 | 0.021 | 0.340 | -1.047 |
| ZBTB34 | 9 | 6606 | 0.369 | 0.361 | 1.697 | 1.898 | 3.385 | 1.599 | 0.041 | 0.390 | -1.404 |
